# Multi-objective optimization of surface finish and VOC emissions in FFF 3D printing using ANN–NSGA-II approach

**DOI:** 10.1038/s41598-025-17304-7

**Published:** 2025-10-02

**Authors:** Mahboob Durai M. A., Denis Ashok S.

**Affiliations:** Cyber Physical Systems Lab, Department of Design and Automation, School of Mechanical Engineering, Vellore Institute of Technology, Vellore, 632014 Tamil Nadu India

**Keywords:** Additive manufacturing, Fused filament fabrication, Process parameter optimization, Multi-objective optimization, NSGA II, Surface roughness, VOC emissions, Engineering, Mechanical engineering

## Abstract

Fused filament fabrication (FFF) is a widely adopted 3D printing technique for the rapid, cost-effective, and customized fabrication of complex microfluidic channels using polylactic acid (PLA), particularly for drug delivery and biomedical applications. However, achieving optimal surface finish and minimizing volatile organic compound (VOC) emissions during printing remains a substantial challenge. The current study proposes a new approach by integrating a hybrid framework of artificial neural networks (ANN) with the non-dominated sorting genetic algorithm II (NSGA-II) to enhance both the quality and environmental safety of the FFF 3D printing process for microfluidic channel applications. The proposed approach optimizes four key process parameters such as layer thickness (LT), print speed (PS), material flow rate (MFR), and raster angle (RA) with a prime objective of obtaining an excellent surface finish with minimum VOC emissions. A customized FFF 3D printer embedded with cost-effective, high-sensitivity emission sensors was designed and developed to facilitate real-time monitoring, thereby offering a unique and economical capability not available in conventional commercial printers. Experimental data generated using central composite design (CCD) were employed to train a high-fidelity ANN model (R² > 95%), which functions as a surrogate model for NSGA-II-based multi-objective optimization. The ANN model demonstrated strong predictive accuracy with low mean squared error (MSE) and high correlation coefficients (R² = 0.9967 for training, 0.956 for validation, and 0.9261 for testing). The proposed ANN-NSGA-II framework identified Pareto-optimal solutions at LT (0.15 mm), PS (40 mm/s), MFR (100%), and RA (30º). The optimization results demonstrate effective control of process parameters, yielding the dual benefit of enhanced surface finish and reduced VOC emissions. The novelty of this work lies in integration of real-time emission sensing with predictive intelligence and evolutionary multi-objective optimization, ensuring high print quality while simultaneously advancing environmentally responsible additive manufacturing (AM) practices.

## Introduction

Fused filament fabrication (FFF) has emerged as a widely adopted additive manufacturing (AM) technology due to its affordability, user-friendliness, and capability to produce complex structures. This method involves the layer-by-layer deposition of thermoplastic materials to create intricate geometries, making it popular across diverse applications^1–3^. In industrial settings, commonly used FFF thermoplastic filaments include acrylonitrile butadiene styrene (ABS)^4^ and polylactic acid (PLA)^5^alongside other materials such as polycarbonate (PC)^6^nylon^7^polyethylene terephthalate glycol (PETG)^8^thermoplastic polyurethane (TPU)^9^polyether ether ketone (PEEK)^10^acrylonitrile styrene acrylate (ASA)^11^high-impact polystyrene (HIPS)^12^and polyvinyl alcohol (PVA)^13^. Each filament type possesses unique properties and functions tailored for specific uses. Among these materials, PLA, a biodegradable polymer derived from renewable resources, has gained significant attention in FFF 3D printing for biomedical and microfluidic applications^14,15^. However, achieving an exceptional surface finish and mechanical properties in PLA-printed components remains a challenge. Critical process parameters such as layer thickness, print speed, print temperature, material flow rate, and raster angle directly govern the surface quality, mechanical integrity, and dimensional accuracy of FFF 3D printed components^16–18^. These parameters influence the deposition dynamics, interlayer bonding, and thermal gradients, all of which are pivotal in determining the functional performance of printed parts^19^.Optimizing these factors is essential in achieving precise geometries, minimizing surface irregularities, and enhancing mechanical reliability, particularly in applications where stringent quality and performance criteria are required such as microfluidic devices and biomedical components^20,21^.

With the desktop FFF 3D printers have gained widespread adoption across various disciplines due to their accessibility and versatility, few studies have found that the 3D printing process releases gas emissions including volatile organic compounds, referred to as VOCs, which are critical to human health when inhaled. Maintaining the functional integrity of printed parts while minimizing the release of VOCs remains a critical challenge, particularly in applications demanding high precision and environmental safety. Hence, there is a need to develop suitable strategies that maintain print quality and emission control to ensure both performance reliability and safer additive manufacturing practices.

Researchers have explored various methods to enhance the surface quality of 3D-printed parts through empirical modeling, including regression analysis coupled with optimization techniques^22^. These strategies are broadly categorized into conventional and advanced approaches. Conventional methods predominantly rely on statistical design of experiments (DOE) techniques such as Taguchi, factorial design, and response surface methodology (RSM), which systematically analyze process parameters to achieve optimal performance^23^. For instance, Wankhede et al.^24^ utilized the Taguchi method to optimize surface roughness and build time for ABS polymers, identifying layer thickness as the most significant factor. A layer thickness of 0.254 mm yielded better surface roughness, while the shortest build time was achieved at 0.3302 mm. Similarly, Altan et al.^18^ employed the Taguchi L16 orthogonal array to study the effects of layer thickness, nozzle temperature, and deposition head velocity on surface roughness and tensile strength of PLA. Their findings indicated that lower layer thickness improved performance, enhancing surface roughness by 12% and tensile strength by 25%. Peng et al.^25^ applied the full factorial method to investigate surface roughness and energy consumption in FFF 3D-printed parts using PLA. They concluded that layer thickness was the most influential factor for both surface roughness and energy consumption. Reddy et al.^26^ utilized the Taguchi method, signal-to-noise (S/N) ratio, and multiple regression analysis to assess surface texture in FFF-printed ABS components. Their study highlighted that layer thickness and build inclination significantly affected surface roughness. Nidagundi et al.^27^ employed the Taguchi orthogonal array and S/N ratio to optimize surface roughness and dimensional accuracy, analyzing layer thickness, orientation, and fill angle using a stylus movement device on cube specimens. Ayrilmis^28^ used Analysis of Variance (ANOVA) to examine the effect of layer thickness on the surface characteristics of wood flour/PLA composites, printing samples at 200 °C across four-layer thicknesses (0.05, 0.1, 0.2, and 0.3 mm). Results showed that surface roughness increased with layer thickness. Overall, a thorough literature review confirms the extensive application of conventional optimization techniques for improving surface roughness in 3D printing. However, it does not capture curvature or provide continuous response surfaces necessary for locating true optima in complex and multi-objective problems whereas the Central Composite Design (CCD) overcomes these limitations.

Conventional optimization techniques often face difficulties in identifying optimal solutions, particularly when numerous parameters influence the process, and are prone to becoming trapped in local minimum. To address these limitations, recent research has increasingly focused on leveraging approximate algorithms to optimize the FFF process. Raju et al.^29^ combined regression modeling with a Taguchi L18 orthogonal array and a hybrid particle swarm optimization-bacterial foraging optimization (PSO-BFO) approach to improve layer thickness, support material, model interior, and orientation for enhanced surface quality and mechanical performance. Their method achieved a 7.44% improvement in general response with limited support material, high-density interior, and a 45°-part orientation. Baskar et al.^30^ validated a mathematical model using ANOVA analysis, confirming statistical significance with p-values below 0.0001. Their approach demonstrated 100% dependability for printing time, 96% for top and bottom layer surface roughness, and 99% for all sides surface roughness. PSO-predicted values showed strong accuracy, with an error margin of less than 4% in experimental comparisons. Pandey et al.^31^ used a multi-criteria genetic algorithm (MCGA) to investigate the effects of part deposition orientation on building time and surface quality, revealing that orientation significantly influences both metrics. Similarly, Tandon et al.^32^ analyzed the interactions of material type, orientation, and infill density through a structured experimental approach. Their multi-response optimization desirability method identified optimal parameters, achieving an average desirability score of 0.84. Asadollahi-Yazdi et al.^33^ employed the non-dominated sorting genetic algorithm (NSGA-II) to optimize layer thickness and orientation angle, reducing material consumption and production duration while maintaining mechanical integrity and surface quality. They found that decreasing layer thickness significantly improved both strength and surface roughness. Chen et al.^34^ applied NSGA-II alongside RSM to enhance tensile strength, surface roughness, and production time for FFF-printed components. Their study analyzed nozzle diameter, liquefier temperature, extrusion velocity, filling velocity, and layer thickness. The integrated approach effectively improved tensile strength minimized surface roughness, and reduced production time. These advancements underscore the potential of approximate algorithms to overcome the limitations of conventional methods and achieve comprehensive optimization in FFF 3D printing.

In addition to combining traditional modeling techniques with approximate algorithm optimization, artificial intelligence (AI) methods such as artificial neural networks (ANN) and adaptive neuro-fuzzy inference systems (ANFIS) offer superior capabilities for predicting optimal manufacturing parameters. These methods surpass conventional approaches in both computational speed and memory efficiency, significantly advancing FFF performance. Jayaseelan et al.^35^ developed a fuzzy system using a hybrid fuzzy-based cross-neighbor filtering (HF-CNF) technique to reduce impulsive and random noise in scanning electron microscope (SEM) images, enhancing image quality while preserving structural details through fuzzy membership functions. Similarly, Yadav et al.^36^ utilized ANFIS to study the effects of extrusion temperature, layer height, and material density on tensile strength. Their findings showed that tensile strength was influenced by extrusion temperature and layer height, with PETG achieving maximum strength at 225 °C and a layer height of 0.1 mm. Sai et al.^37^ optimized FFF process parameters to enhance surface quality, reduce build time, and increase compressive strength for biomedical implants. Using ANFIS combined with the whale optimization algorithm (WOA), optimal parameters were identified, achieving a surface roughness of 2.756 μm, a build time of 84.42 min, and a compressive strength of 14.279 MPa. Saad et al.^38^ demonstrated the integration of ANN with the symbiotic organism search (SOS) algorithm to optimize the FFF process and improve surface roughness. The optimized 4-8-8-1 ANN architecture produced accurate predictions, outperforming traditional RSM approaches. The success of ANN-SOS underscores its potential for broader applications in additive manufacturing.

Surface roughness in FFF 3D printing significantly influences the quality of fabricated components, particularly in microfluidic applications. This study focuses on optimizing FFF technology for microfluidic channels, where surface roughness plays a critical role in affecting fluid flow characteristics such as resistance, mixing efficiency, and potential leakage. Variations in roughness result from parameters like layer height, nozzle diameter, infill density, and print orientation relative to the build layers^39^. For instance, channels aligned with printing layers tend to exhibit smoother surfaces compared to perpendicular orientations, impacting hydraulic performance. Material choice also influences surface quality; PLA + demonstrates superior smoothness and dimensional accuracy relative to ABS^40^. Addressing these factors is crucial for optimizing design and operational parameters in microfluidic applications, ensuring reliable fluid transport within 3D-printed systems. Despite its importance, few studies have specifically optimized FFF 3D printing for microfluidic channels, highlighting a need for further research to refine printing strategies and enhance surface quality for improved performance and broader application of 3D-printed microfluidic devices.

Moreover, heating PLA filament during the FFF process generates particulate matter (PM) and volatile organic compounds (VOCs). The emission rates depend on extrusion temperature, filament composition, and print speed. These pollutants pose health risks, such as respiratory irritation, and contribute to environmental concerns by degrading indoor air quality^41^. Therefore, understanding and controlling VOC emissions are essential for ensuring safe and sustainable additive manufacturing practices. Research in emission control and health monitoring of 3D printers has significantly advanced over time. Early studies by Stephens et al.^42^ revealed that lactide, a byproduct of PLA degradation, is emitted at higher rates from PLA filaments, ranging from 4 to 5 µg/min. Analytical techniques like gas chromatography (GC), scanning mobility particle sizer (SMPS), and condensation particle counter (CPC) were employed to characterize PM and VOC emissions, emphasizing the need for effective emission control measures in 3D printing. Kim et al.^43^ showed that PLA filaments release ultrafine particles (UFPs, 1–100 nm) and trace quantities of hazardous VOCs such as lactide, formaldehyde (HCHO), and acetaldehyde, quantified using SMPS techniques. Similarly, Byrley et al.^44^ investigated emissions using thermal desorption and mass spectrometry (MS) techniques, detecting UFPs and VOCs like styrene and ethylbenzene. S Ding et al.^45^ assessed UFP and VOC emissions across different filaments, finding emission peaks during liquefaction, with VOC yields of 0.03% (PLA), 0.21% (ABS), and 2.14% (PVA). Their study suggested optimizing heating rates and implementing emission control measures for enhanced safety. Singh et al.^46^ explored VOC and UFP emissions in enhanced PLA filaments, emphasizing the influence of additives on emission levels and advocating for safer filament formulations and improved emission controls in consumer environments. Similarly, Davis et al.^47^ demonstrated that emissions vary based on filament type, temperature, and degradation, utilizing GC and MS techniques to address health risks in poorly ventilated spaces. Their findings promote safer practices through better ventilation, filtration, and low-emission materials. The correlation between rising temperatures and VOC emissions, underscoring the health hazards posed in inadequately ventilated areas was further highlighted^41^.

It is found that extensive research has been conducted on the FFF 3D printing process for optimization of process parameters through various methodologies to achieve precise geometric design. However, the emission safety of the FFF 3D printing process and geometric surface finish optimization for microfluidic channel applications remain underexplored. This study tackles these challenges by introducing a novel 3D printer equipped with advanced sensors for emission monitoring during the 3D printing of parts. A novel approach involving artificial neural networks (ANNs) and the non-dominated sorting genetic algorithm II (NSGA-II) is proposed to identify Pareto-optimal solutions that simultaneously enhance surface quality and promote environmental sustainability. Through the integration of AI-driven predictive modeling with metaheuristic optimization, this study effectively bridges existing gaps in the optimization of the FFF process, offering a pathway for achieving functional performance and environmental safety of microfluidic channels.

## Experimental setup and design

### Experimental setup

**Fig. 1:**
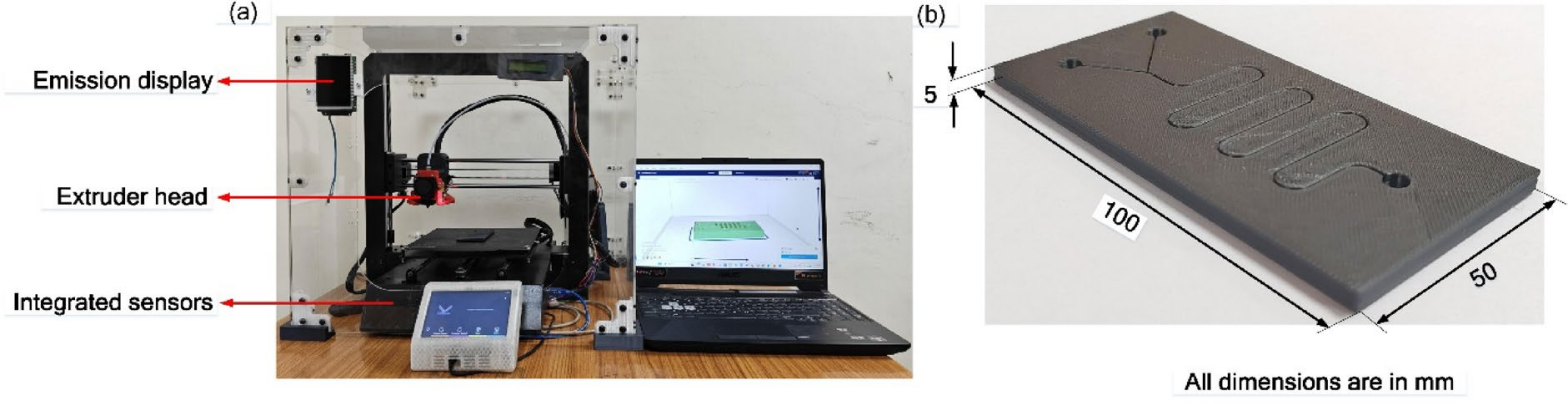
(**a**). Experimental setup of the customized FFF 3D printer, (**b**). 3D-printed microfluidic channel.

In this study, a customized FFF 3D printer was assembled in-house to provide enhanced flexibility for process parameter control tailored to experimental requirements. The printer features a build volume of 200 × 200 × 210 mm^3^ and employs an extruder capable of reaching temperatures up to 260 °C with a heated build platform maintained up to 80 °C. The system is enclosed within a custom-built chamber to ensure controlled environmental conditions during 3D printing process. Additionally, the printer is integrated with high-sensitivity, cost-effective electrochemical sensors (AGS02MA and DFRobot Fermion) for real-time monitoring of VOC emissions, enabling simultaneous assessment of surface finish and emission characteristics. This bespoke configuration allows for comprehensive mechanical and environmental performance evaluation beyond the capabilities of standard commercial 3D printers. In this study, specimens were fabricated using standard FFF protocols, without adherence to specific ASTM printing standards. Surface roughness was measured via Keyence VHX-7000 microscope, following ISO 4287 for texture characterization. Moreover, the fused filament fabrication was conducted in line with best practices outlined in ISO/ASTM 52,900 for additive manufacturing terminology and processes. Figure 1 (a) shows the experimental setup of customized FFF 3D printer and (b) shows the 3D-printed microfluidic channel, while Table 1 outlines the specifications of the customized FFF 3D printer.

**Table 1:** Specifications of the customized FFF 3D printer.

Parameters	Specifications	Units/Range
Material type supported	PLA, ABS, PETG, TPU	-
Build size	200 × 200 × 210	mm^3^
Filament diameter	1.75	mm
Nozzle diameter	0.4	mm
Extruder temperature	Recommended = 200, Maximum = 260	°C
Printing speed	120	mm/s
AGS02MA	Metal oxide semiconductor (MOS) gas sensor	0–9999 ppb, resolution 1 ppb.
DFRobot Fermion	MEMS metal oxide semiconductor (MOS) gas sensor	0.1–10,000 ppm

**Fig. 2:**
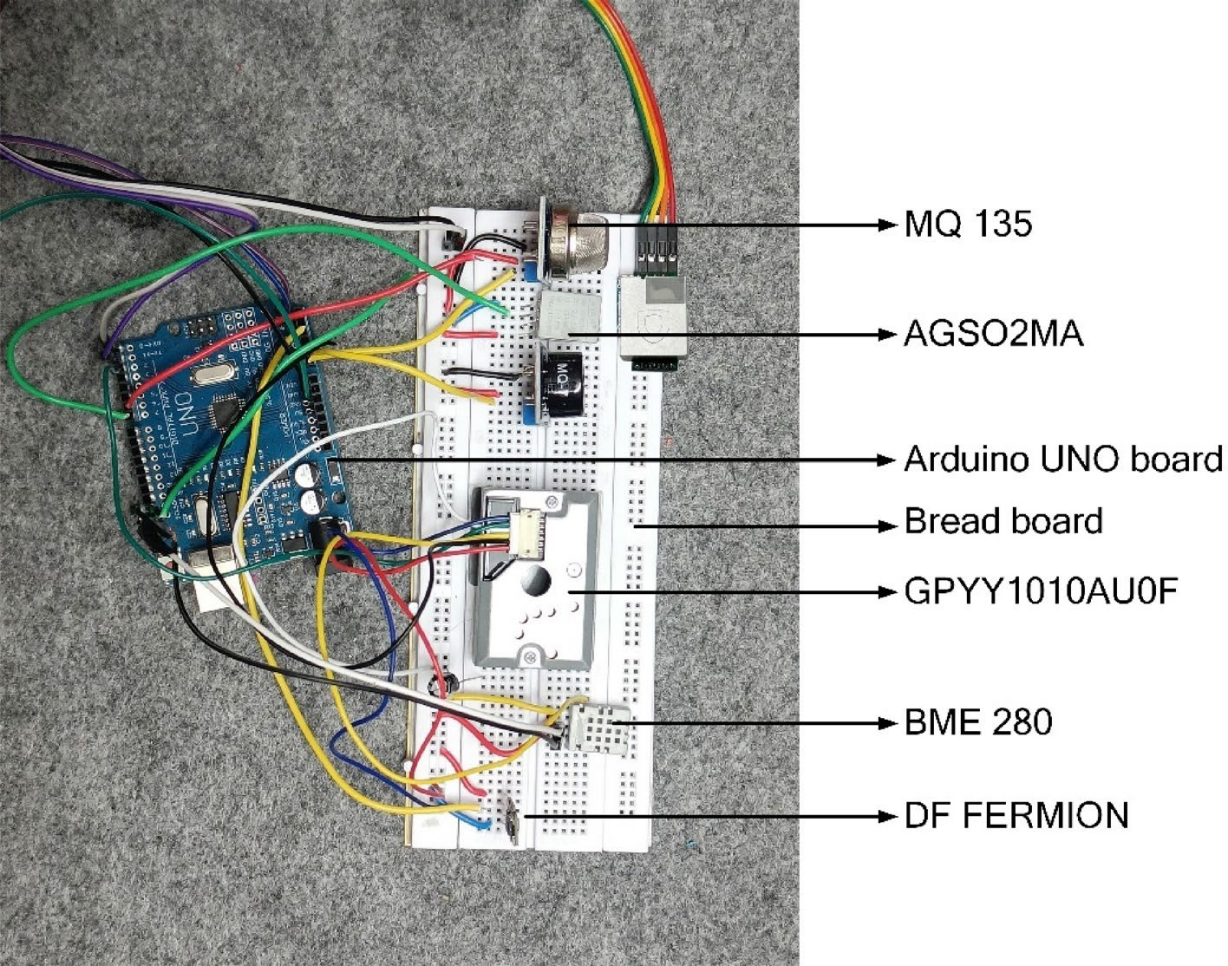
Sensor integration for emission monitoring in FFF 3D printing setup.

Figure 2 illustrates the different sensors integrated into the FFF 3D printer. In this study, two distinct VOC sensors were utilized for emission assessment during the FFF 3D printing process. The AGS02MA, a metal oxide semiconductor (MOS) gas sensor used in this study was factory calibrated, offering stable digital I2C output with an accuracy of ± 15% at standard conditions. It is specifically designed for indoor TVOC monitoring and offers a detection range of 0-9999 ppb with a fine resolution of 1 ppb, making it well suited for detecting low-concentration VOC trends. Complementing this, the DFRobot Fermion MEMS MOS gas sensor with a detection range of 0.1–10,000 ppm range was employed to measure higher VOC concentrations, including formaldehyde (HCHO). This sensor exhibits high sensitivity, enabling real time detection of VOC spikes and cumulative exposure levels during 3D printing.

It is acknowledged that environmental factors such as temperature, humidity, ventilation, and background air pollutants can influence sensor response, as MOS-based VOC sensors rely on adsorption-desorption mechanisms and are sensitive to such variations. However, this experimental design emphasizes on capturing relative emission trends under different process parameters, which remain valid for these influences.

Furthermore, controlled environment experiments were conducted within an enclosed chamber equipped with a ventilation blower, ensuring the standardized airflow conditions. These tests confirmed that the observed patterns of particle and VOC emissions remain consistent and are not significantly influenced by differing ventilation conditions inherent to various testing sites. It is important to note that the present study does not report large-scale building dimensions or operational ventilation characteristics, as the experiments were deliberately conducted in a standardized enclosed chamber of 10 ft X 10 ft with controlled HVAC settings of 24 °C and relative humidity of 90%. This controlled configuration was chosen to minimize environmental variability and ensure consistent repeatability of emission measurements, thereby prioritizing well-defined boundary conditions over generalized building airflow modelling, which lies beyond the intended scope of this study.

### Materials and methods

This research employs polylactic acid (PLA) polymer, one of the most widely used materials in FFF 3D printing alongside acrylonitrile butadiene styrene (ABS), due to its accessibility, versatility in manufacturing, and cost-effectiveness. PLA is a biodegradable polymer derived from renewable resources, characterized by its high rigidity, low warping, minimal nozzle clogging, and ease of printing. With a low melting point (190–220 °C), PLA demands less bed heating, contributing to reduced energy consumption^48^. It is particularly suited for prototyping, education, medical applications, and low-load uses, though its brittleness and low heat resistance limit its scope. Unlike ABS, PLA emits minimal VOCs, offering a more environmentally friendly option. Table 2 shows the material properties of the PLA filament^49^.

**Table 2:** Material properties of the PLA filament.

Properties	Specifications
Melting temperature	190–220 °C
Bed temperature	40–60 °C
Glass transition temperature (Tg)	~ 60 °C
Tensile strength	50–70 MPa
Young’s modulus	2.7–16 GPa
Density	1.24 g/cm³
Filament diameter	1.75 mm
Thermal conductivity	~ 0.13–0.25 W/(m.K)

This study aligns the selection of process parameters to minimize VOC emissions while enhancing surface quality. The key parameters include layer thickness (LT), printing speed (PS), material flow rate (MFR), and raster angle (RA). Thus, optimizing input process parameters is essential to achieving improved surface quality with reduced VOC emissions.

### Specimen fabrication

The operational performance of the customized FFF 3D printer was evaluated by designing (20 × 20 × 3) mm cubes using Autodesk Fusion 360 and manufactured through the fused filament fabrication (FFF) process. The 3D CAD model was converted into a stereolithography (.stl) file, providing a triangulated representation of the cube’s surface geometry. This.stl file was subsequently imported into Cura for slicing, and the generated G-code was loaded into the customized FFF 3D printer. The printer extruded thermoplastic filament in successive layers to fabricate the cube. Surface roughness (R_a_), formaldehyde (HCHO), and total volatile organic compound (TVOC) emissions were systematically analyzed under varying printing parameters^50^including layer thicknesses (LT) of 0.15 mm, 0.20 mm, and 0.25 mm; printing speeds (PS) of 40 mm/s, 50 mm/s, and 60 mm/s; material flow rates (MFR) of 98% 100% and 102% and raster angles (RA) of 30°, 60°, and 90°. Some variation in VOC readings may have occurred due to environmental factors, but the comparisons between different process parameters remain consitent and reliable. The selected parameters, including layer thickness (LT), printing speed (PS), material flow rate (MFR), and raster angle (RA), are detailed in Table 3.

**Table 3:** Different levels of print process parameters.

Parameter	Symbol	Level 1	Level 2	Level 3	Units
Layer thickness	LT	0.15	0.20	0.25	mm
Print speed	PS	40	50	60	mm/s
Material flow rate	MFR	98	100	102	%
Raster angle	RA	30	60	90	Degree

### Specimen Preparation and characterization

**Fig. 3:**
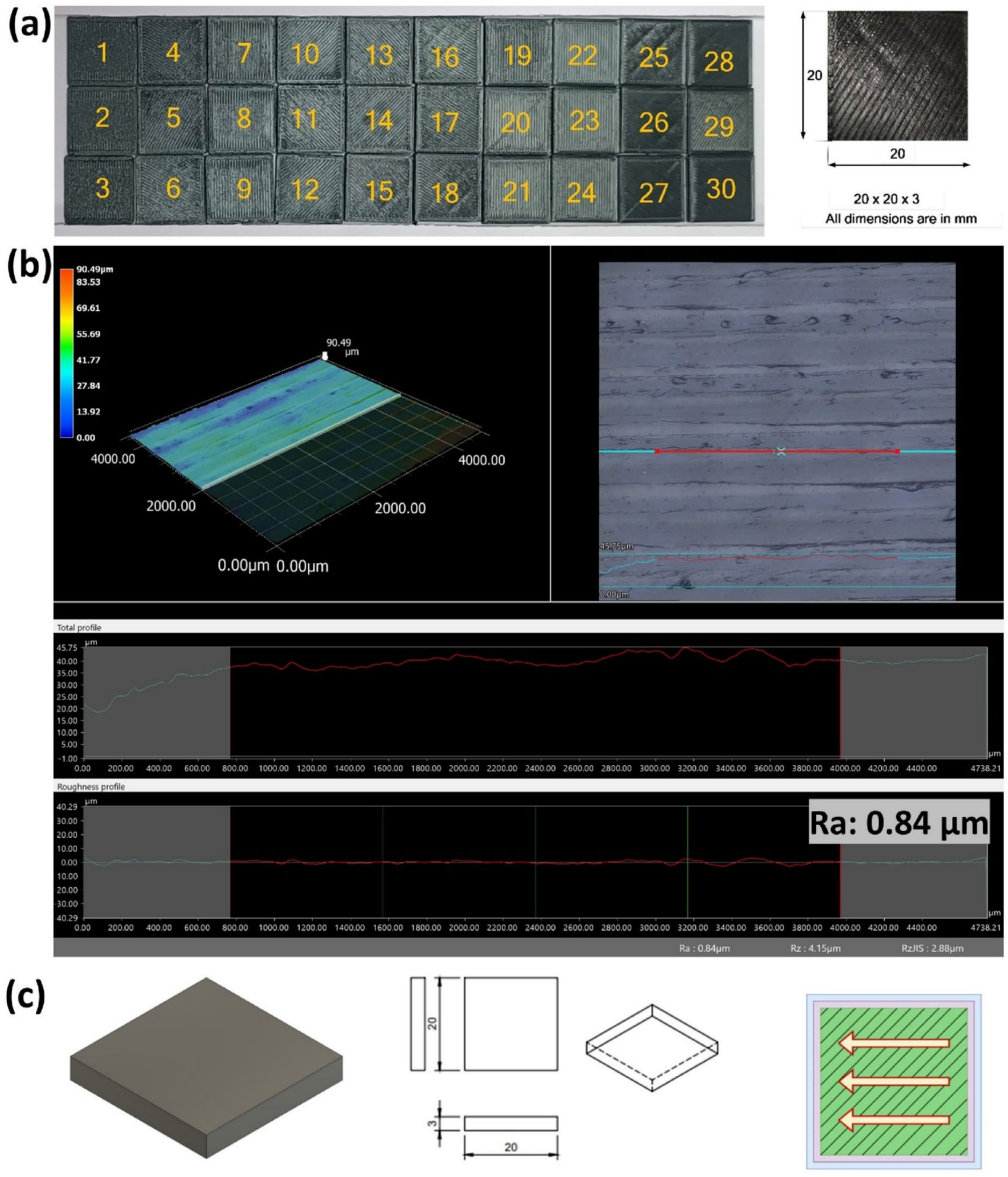
(**a**). Top view of PLA test specimens. (**b**). Surface roughness measurement. (**c**). Specimen geometry and scan direction.

To facilitate a comparative evaluation of surface quality across the parameter matrix, all 30 printed specimens were organized into a structured layout. Each specimen represents a distinct parameter combination from the central composite design (CCD) matrix. Figure 3 (a) presents a top-view arrangement of the specimens in a matrix format. This systematic spatial distribution not only aids in visual differentiation of surface textures influenced by varying parameters but also streamlines the inspection process during analysis.

The layout was specifically designed to highlight gradational changes in surface finish attributable to different combinations of LT, PS, MFR, and RA. This spatial methodology provided a direct visual and analytical correlation between process settings and observed surface characteristics, thereby reinforcing the robustness of the experimental design and evaluation protocols.

The specimen dimensions and the direction of measurement are depicted in Fig. 3(c). The surface roughness (R_a_) of the top surface of a PLA-printed component was evaluated across different parameter configurations, with average roughness values obtained using a Keyence 3D non-contact microscope. Figure 3(b) illustrates the surface roughness measurement and analysis for a representative specimen. The impact of four critical FFF process parameters on surface roughness and VOC emissions was thoroughly examined.

### Design of experiments and data collection

A systematic investigation was conducted using a rotatable Central Composite Design (CCD), based on the principles of Design of Experiments (DoE), to facilitate experimental data collection. The experimental setup comprised 30 runs, including 16 factorial points, 8 axial (star) points, and 6 center points. To model the functional relationships between process parameters and response variables, surrogate modeling techniques such as RSM and ANN were employed^51^. These predictive models were subsequently utilized as objective functions in the optimization process to achieve enhanced outcomes.

Additionally, numerical readings (in mg/m³) for formaldehyde (HCHO) and total volatile organic compounds (TVOC) were obtained using AGS02MA and DFRobot Fermion electrochemical sensors. The corresponding VOC emission values are presented in Table 4.

**Table 4:** Performance evaluation of CCD experimental design.

Run	FFF Process parameters				Experimental result		
	Factor 1	Factor 2	Factor 3	Factor 4	Response 1	Response 2	Response 3
	LT	PS	MFR	RA	*R*_a_	HCHO	TVOC
	(mm)	(mm/s)	(%)	(Degrees)	(µm)	(mg/m^3^)	(mg/m^3^)
1	0.15	40	98	30	3.25	0.003	0.071
2	0.25	60	98	90	5.51	0.0044	0.1003
3	0.2	50	100	60	4.54	0.0031	0.0841
4	0.2	50	100	60	4.54	0.0031	0.0841
5	0.25	40	102	90	5.29	0.0037	0.08
6	0.25	40	102	30	5.29	0.0036	0.0884
7	0.25	60	102	30	7.14	0.0043	0.1161
8	0.15	60	98	30	5.03	0.0039	0.0987
9	0.2	50	98	60	4.88	0.004	0.0988
10	0.2	50	100	60	4.54	0.0031	0.0841
11	0.2	50	100	90	4.62	0.004	0.0987
12	0.15	50	100	60	2.33	0.0029	0.0803
13	0.15	60	98	90	3.48	0.0029	0.0803
14	0.25	60	98	30	6.52	0.0042	0.1002
15	0.25	50	100	60	6.1	0.0039	0.0908
16	0.2	50	100	60	4.54	0.0031	0.0841
17	0.25	60	102	90	7.19	0.0045	0.105
18	0.2	60	100	60	5.41	0.0037	0.101
19	0.15	40	102	30	0.84	0.0028	0.0608
20	0.15	60	102	30	3.6	0.003	0.088
21	0.25	40	98	30	5.64	0.0035	0.089
22	0.15	40	98	90	2.31	0.003	0.0802
23	0.25	40	98	90	5.6	0.0038	0.0885
24	0.2	50	102	60	4.5	0.0039	0.0987
25	0.2	50	100	60	4.54	0.0031	0.0841
26	0.2	40	100	60	4.38	0.0034	0.0881
27	0.2	50	100	60	4.54	0.0031	0.0841
28	0.15	40	102	90	1.71	0.0032	0.0802
29	0.2	50	100	30	4.11	0.0033	0.0881
30	0.15	60	102	90	4	0.003	0.0881

### Mathematical model for surface roughness, HCHO, and TVOC

The values in Eq. 1 for the response variable such as R_a_, HCHO, and TVOC emissions were derived using a systematic Response Surface Methodology (RSM) approach based on a Central Composite Design (CCD). The design matrix consisted of 30 experimental runs with coded values for four process parameters namely LT, PS, MFR, and RA. The general form of the second-order RSM model is expressed as:

1 $$\:\text{Y}={\beta\:}_{0}+{\beta\:}_{i}{x}_{i}+{\beta\:}_{ii}{x}_{i}^{2}+{\beta\:}_{ij}{x}_{i}{x}_{j}+\epsilon\:$$

Expanded for the four process parameters: LT, PS, MFR, and RA,

2 $$\begin{aligned} &\:\text{Y}={\beta\:}_{0}+{\beta\:}_{1}{x}_{1}+{\beta\:}_{2}{x}_{2}+{\beta\:}_{3}{x}_{3}+{\beta\:}_{4}{x}_{4}+\:{\beta\:}_{11}{x}_{1}^{2}+{\beta\:}_{22}{x}_{2}^{2}+{\beta\:}_{33}{x}_{3}^{2}+{\beta\:}_{44}{x}_{4}^{2}+{\beta\:}_{12}{x}_{1}{x}_{2}\\&\qquad\qquad+\:{\beta\:}_{13}{x}_{1}{x}_{3}+{\beta\:}_{14}{x}_{1}{x}_{4}+{\beta\:}_{23}{x}_{2}{x}_{3}+{\beta\:}_{24}{x}_{2}{x}_{4}+{\beta\:}_{34}{x}_{3}{x}_{4}\\&\qquad\qquad+\epsilon\:\end{aligned}$$

In this model, Y represents the response variable (Ra, HCHO, or TVOC), x₁, x₂, x₃, x₄ denote the coded values for LT, PS, MFR, and RA respectively and ε represents random error term.

The regression coefficients (β) in the second-order polynomial model were estimated through the least squares method as expressed by:

$${\rm \beta = (X^{\prime}X)^{-1}X^{\prime}Y.}$$

Where:

- X is the 30 × 15 design matrix containing coded parameter values and their interactions.
- Y is the 30 × 1 response vector for each output variable.
- β is the 15 × 1 coefficient vector.

This modelling approach effectively captures the nonlinear relationships between process factors and performance outcomes, providing a robust foundation for optimization and predictive analysis.

3 $$\begin{aligned}&{R}_{a}\:=539.35-\:(242.72*LT-\:(1.74*PS (908.26*MFR-(0.47*RA)\\&-(0.43*LT*PS)+(347.50*LT*MFR)+(0.0092*LT*\:RA)\\&+(1.58*PS* \:MFR)-(0.0004*\:PS*RA)+(0.51*MFR*RA)\\&-[132.25(LT^{2}]+\:\:[0.0035{(PS}^{2}]+[360.96{(MFR}^{2}]-[0.0002\:{(RA}^{2}]\end{aligned}$$

4 $$\begin{aligned}\:HCHO\:=&0.9808-\:\left(0.0377*LT\right)+\:\left(0.0002*PS\right)-\:\left(1.95*MFR\right)-\left(0.0001*\:RA\right)+\\ &\left(0.0593*LT*MFR\right)+\left[\left(5.46{\text{e}}^{-5}\right)*LT*\:RA\right]-\left(0.0002*PS*MFR\right)-\\ &\left[\left(2.90{\text{e}}^{-7}\right)*PS*RA\right]+\left(0.0001*MFR*RA\right)-[0.0707\left(LT\right)^{2}]-\\ &[\left(4.84{{\text{e}}^{-7})\left(PS\right)}^{2}\right]+\left[0.97{\left(MFR\right)}^{2}\right]+\left[\left(9.35{\text{e}}^{-8}\right){\left(RA\right)}^{2}\right]\end{aligned}$$

5 $$\begin{aligned}\:TVOC=&\:14.80-\:\left(2.90*LT\right)-\:\left(0.03*PS\right)-\:\left(27.23*MFR\right)-\left(0.0047*RA\right)-\\ &\left(0.0051*LT*PS\right)+\left(4.91*LT*MFR\right)-\left(0.0035*LT*\:RA\right)+\\ &\left(0.03*PS*MFR\right)-\left[\left(\:2.14{\text{e}}^{-5}\right)\left(PS*\:RA\right)\right]+\left(0.007*MFR*RA\right)-\\ &[3.34\left(LT\right)^{2}]+[\left(6.49{\text{e}}^{-6}\right){\left(PS\right)}^{2}]+[12.13{\left(MFR\right)}^{2}]-[\left(5.50{\text{e}}^{-7}\right){\left(RA\right)}^{2}\end{aligned}$$

“.

These regression equations define the functional relationship between these critical printing parameters for a PLA-printed component. These equations include a regression coefficient, linear, quadratic, and interaction terms for LT, PS, MFR, and RA. The coefficient before each term shows the nature of its influence: a positive coefficient represents a positive effect, while a negative coefficient denotes a negative impact. Once the surrogate models are established, they can be seamlessly incorporated into the multi-parameter process optimization (MPPO) framework. The general MPPO formulation for optimizing R_a_, HCHO, and TVOC based on the process parameters in the FFF process is outlined as follows.

**Table:** 

Objective functions
Minimize Surface roughness (R_a_)
Minimize Formaldehyde emissions (HCHO)
Minimize total volatile organic compounds emissions (TVOC)
Subject to:
FFF process parameter constraints: −1 ≤ x_i_ ≤ 1, i = 1,2,3,….,n

where xi represents the normalized values of the process parameters (LT, PS, MFR and RA).

ANOVA results indicate that for surface roughness (R_a_), LT and PS are statistically significant factors (*p* < 0.005), with corresponding F-values of 87.01 and 17.80, respectively. In contrast, MFR (*p* = 0.992) and RA (*p* = 0.858) were insignificant within the tested ranges, indicating it had a minimal impact on R_a_.

### Methodology

**Fig. 4:**
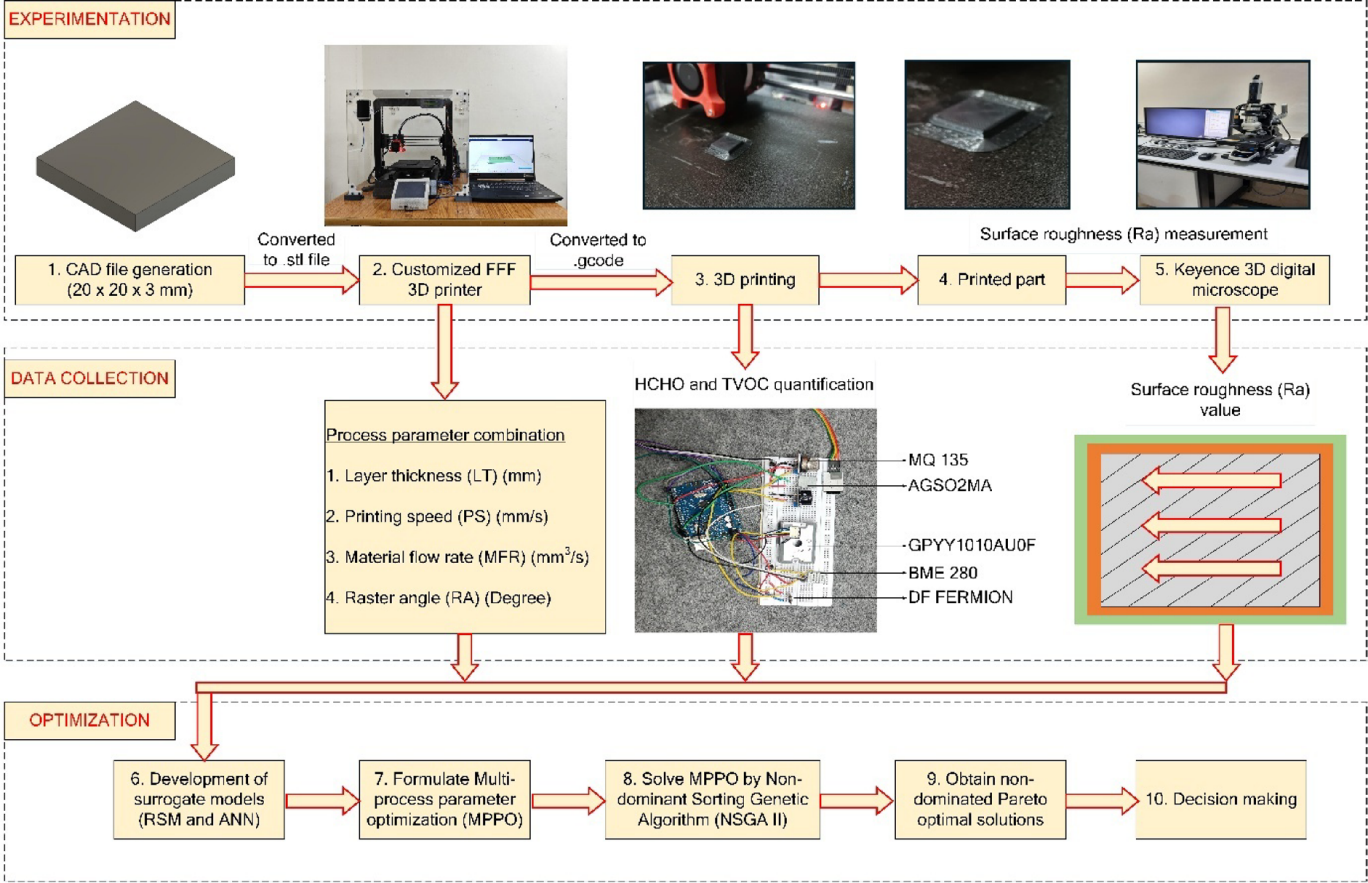
Experimental workflow for FFF 3D printing.

A CAD model (20 × 20 × 3 mm) was generated and converted to.stl, then.gcode format for 3D printing on a customized FFF printer. Key process parameters such as layer thickness, printing speed, material flow rate, and raster angle were varied experimentally. The printed part’s surface roughness (R_a_) was measured using a Keyence 3D digital microscope, while HCHO and TVOC emissions were quantified by sensor array. Surrogate models (RSM, ANN) were developed, and multi-process parameter optimization was formulated and also represented in Fig. 4. The NSGA-II algorithm yielded Pareto optimal solutions, guiding decision-making for improved print quality and lower emissions.

## Development of ANN predictive model using BPNN

### Model architecture and design

**Fig. 5:**
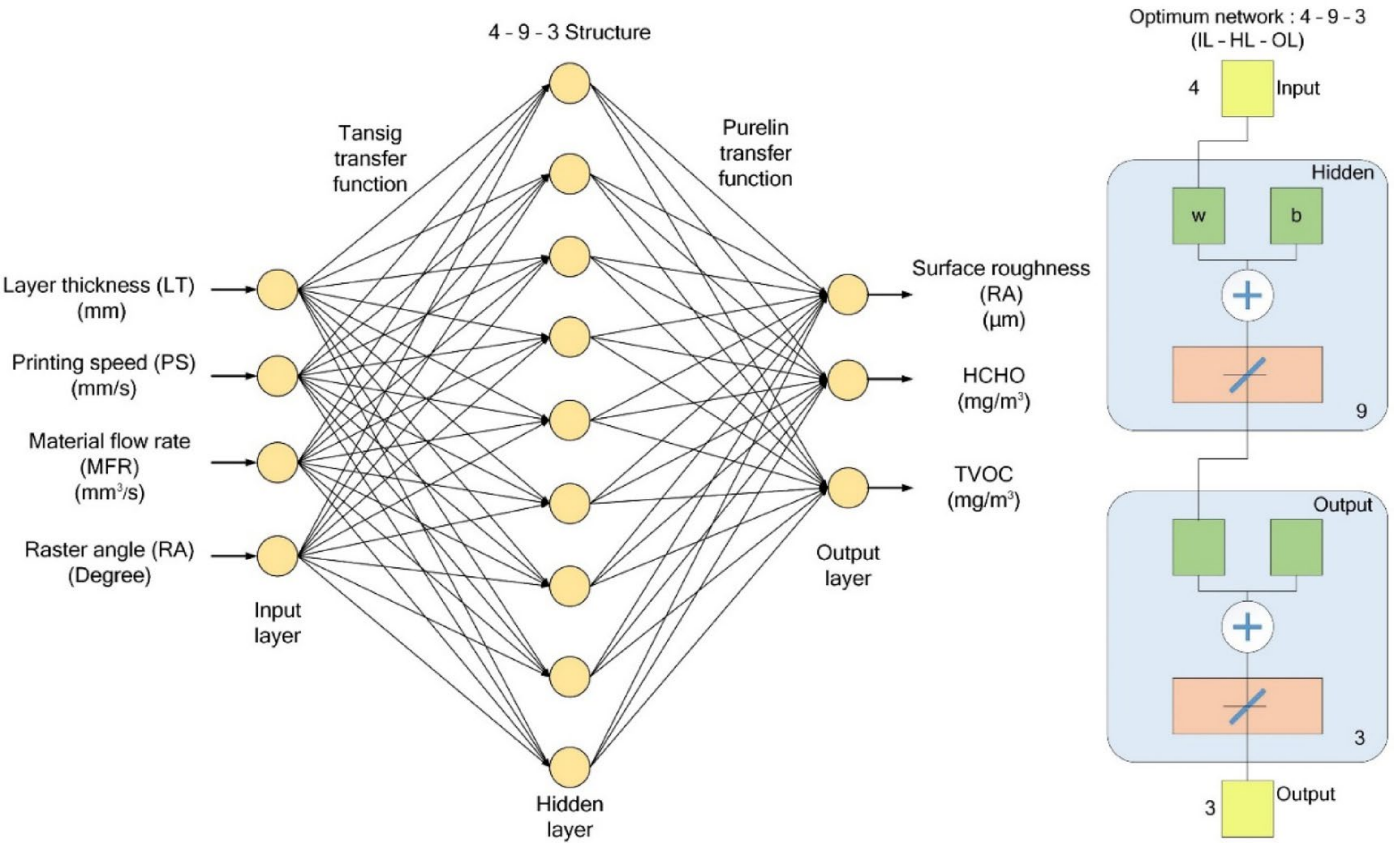
The ANN architecture.

Artificial neural networks (ANNs) offer an innovative methodology for addressing complex problems by correlating nonlinear relationships within multidimensional data. They excel in constructing multivariable systems with intricate interactions through data-driven learning processes. Inspired by the signal processing of biological neurons, an ANN consists of an input layer, one or more hidden layers, and an output layer. Each neuron is connected and assigned a weight and bias value. To produce the predicted output, weights are combined with the bias and processed through an activation function. The specific ANN model in this study was developed using MATLAB, employing a 4-9-3 network configuration, as depicted in Fig. 5. The input layer comprises four neurons representing the process variables (LT, PS, MFR, and RA). The hidden layer contains nine neurons with Tansig activation functions, enabling nonlinear mapping. The output layer includes three neurons (R_a_, HCHO, and TVOC), which utilize a Purelin activation function to generate continuous numerical outputs corresponding to the response variables. This ANN structure, built upon mathematical functions, presents a robust alternative to conventional approaches, particularly for real-world problems involving complex differential equations. The backpropagation neural network (BPNN) method, a recurrent gradient-based algorithm, is employed to minimize the mean squared error (MSE) between actual and desired outputs. To implement the computational workflow described above, Design-Expert^®^ 2018 was used to generate the Central Composite Design (CCD) matrix and to perform statistical analyses, including parameter coding, response surface methodology (RSM) model development, and Analysis of Variance (ANOVA) validation. The artificial neural network (ANN) was implemented in MATLAB R2021a using the Neural Network Toolbox with a 4-9-3 architecture and Levenberg-Marquardt backpropagation. Multi-objective optimization via the non-dominated sorting genetic algorithm II (NSGA-II) was executed using MATLAB’s Global Optimization Toolbox. These software platforms provided a robust and efficient environment for model development, training, and optimization.

### Backpropagation neural network (BPNN) learning algorithm

**Fig. 6:**
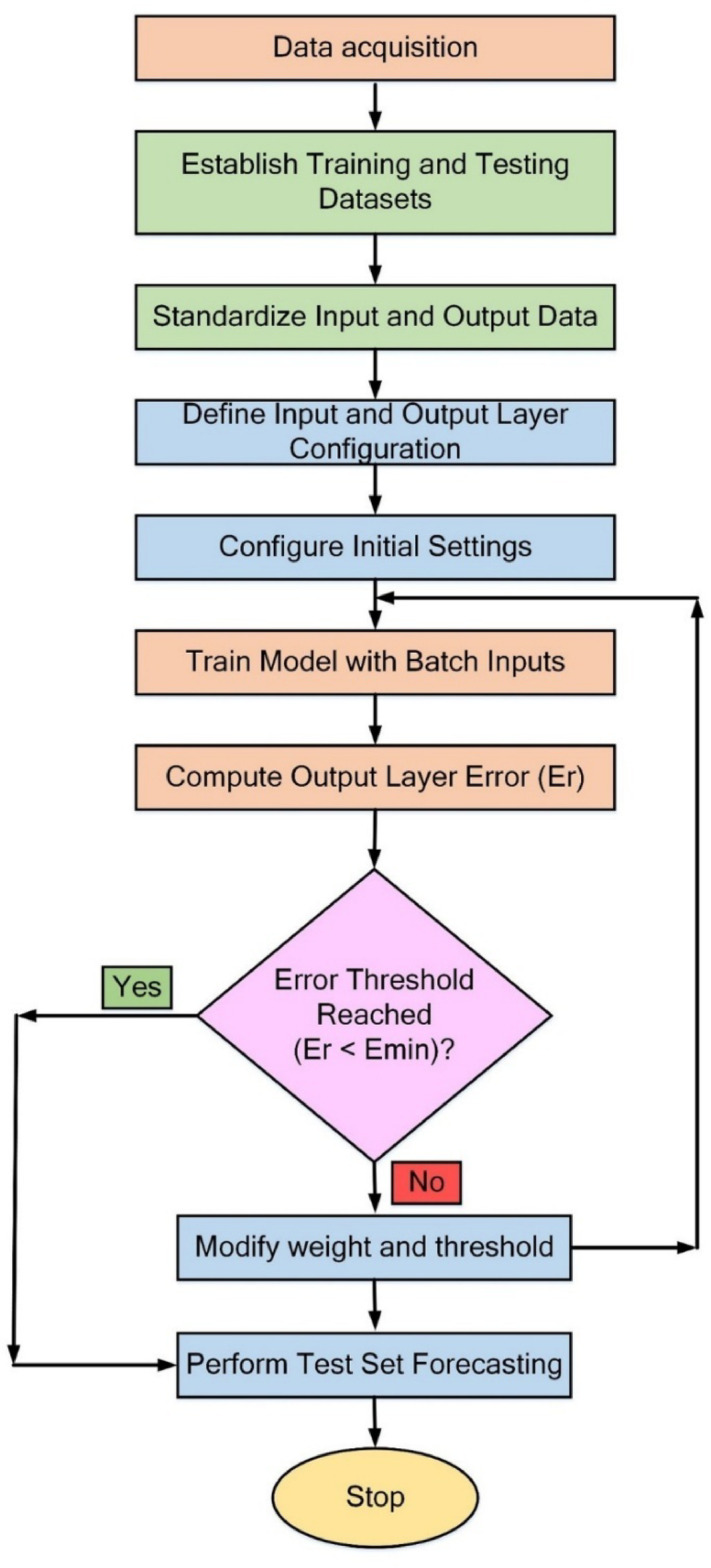
BPNN learning algorithm.

Figure 6 depicts the backpropagation (BP) learning algorithm. It is commonly a three-layered neural network consisting of an input layer, a hidden layer, and an output layer. The process begins with data acquisition, where input and output datasets are gathered and prepared. The dataset is then partitioned into training and testing subsets to ensure a representative sample for model learning and validation. Subsequently, the input and output data are standardized within a consistent range to facilitate model convergence. The configuration of the input and output layers is defined by specifying the number of neurons and selecting suitable activation functions, such as Tansig for hidden layers and Purelin for output layers. Initial settings, including weight initialization, learning rate, error goal, and maximum epochs, are established to guide the training process. During training, batch inputs are utilized, and forward propagation generates predictions. The error at the output layer (E_r_) is calculated by comparing predicted outputs with actual values, often using performance metrics such as MSE.

At each iteration, the model evaluates whether the error threshold (E_min_) has been achieved. If the error falls below the desired threshold, the model advances to test set forecasting to assess its performance on unseen data. If the error remains above the acceptable range, the model adjusts weights and biases using BP and continues training. This iterative process continues until the error threshold is satisfied. Upon reaching this threshold, the model undergoes final evaluation using the test dataset. This comprehensive approach ensures the development of a well-trained ANN model with enhanced predictive performance, accuracy, generalization, and reliability for forecasting tasks. Table 5 outlines the parameters allocated for training the ANN. These training factors were meticulously selected to enhance both the performance and accuracy of the ANN model. The hidden layer incorporates 9 neurons, striking a balance between model complexity and computational efficiency, ensuring sufficient learning capacity without the risk of overfitting. The choice of 1000 training epochs with a maximum limit of 5000 was based on a convergence analysis performed on our limited dataset of 30 samples (20 training, 5 validations, and 5 testing). Given the small size of the validation set, implementing an early stopping criterion often results in high variance and unreliable convergence signals, which may prematurely halt the training before the model achieves optimal performance. To address this, we employed a Sum of Squared Errors (SSE) goal of 0.01 as a more stable convergence criterion suitable for small datasets. The fixed epoch approach ensured consistency and reproducibility across training runs without the artifacts introduced by early stopping in limited data scenarios.

**Table 5:** Specifications of the training parameters.

Parameters for ANN training	Values
No. of neurons in the hidden layer	9
Epochs	1000
Maximum number of epochs	5000
Maximum validation set	15%
Sum squared error goal	0.01

The experimental data distribution is explained with 70% allocated for training, 15% for validation, and 15% reserved for testing. The ANN model underwent additional validation and testing to determine its prediction accuracy and error percentage. A lower MSE value reflects improved predictive performance for surface roughness (R_a_), total volatile organic compounds (TVOC), and formaldehyde (HCHO). This ANN model serves as a computational tool designed to optimize FFF 3D printing by minimizing the need for trial-and-error experimentation, enhancing surface quality, and reducing harmful emissions.

Performance metrics used:

6 $$\:{R}^{2}=1-\:\frac{{\sum\:}_{i=1}^{n}{\left({\text{y}}_{\text{i},\text{p}}-{y}_{i,e}\right)}^{2}}{{\sum\:}_{i=1}^{n}{\left({\text{y}}_{\text{i},\text{p}}-{y}_{e,\:\:avg}\right)}^{2}}\:$$

7 $$\:MSE=\:\frac{{\sum\:}_{i=1}^{n}{\left({\text{y}}_{\text{i},\text{e}}-{y}_{i,p}\right)}^{2}\:}{n}\:$$

8 $$\:MAE=\:\frac{{\sum\:}_{i=1}^{n}\left({\text{y}}_{\text{i},\text{e}}-{y}_{i,p}\right)\:}{n}\:$$

where y_i, e_, y_i, p_, y_e, avg_, and n are the experimental value, predicted value, average experimental value, and the number of data, respectively. The evaluation of the ANN model’s accuracy and predictive capability was conducted using statistical metrics, including the coefficient of determination (R²), mean squared error (MSE), and mean absolute error (MAE), as defined in Eqs. (5–7).

Table 6 summarizes the distribution of observations across the training, validation, and testing phases, alongside the respective MSE and R² values. During the training phase, which included 20 observations, the ANN model achieved a remarkably low MSE of 0.0446 and a high R² value of 0.9967, signifying an excellent fit and a strong relationship between the predicted and actual values. This demonstrates the model’s proficiency in capturing the underlying patterns within the training dataset. In the validation phase, consisting of 5 observations, the model recorded a higher MSE of 0.6491 and a slightly reduced R² value of 0.9568, reflecting reasonable generalization capabilities. While the error increased compared to the training phase, the model continued to demonstrate a high degree of predictive accuracy, effectively generalizing to unseen data. The testing phase, also involving 5 observations, showed the highest MSE of 0.9212 and an R² value of 0.9271, indicating a slight drop in performance when evaluated on independent test data. Nonetheless, the model maintained a strong R² value, signifying reliable prediction capabilities while accounting for minor deviations in the test data. The consistently low MSE and high R² values across all phases confirm that the ANN model is well-trained, exhibits robust predictive accuracy, and demonstrates effective generalization across both validation and testing datasets.

**Table 6:** Training, validation and test distribution of the observations.

	Observations	MSE	*R* ^2^
Training	20	0.0446	0.9967
Validation	5	0.6491	0.9568
Testing	5	0.9212	0.9271

It is acknowledged that the correlation coefficient obtained for testing dataset (*R* = 0.9271) is lower than that for the training (*R* = 0.9967) and validation (*R* = 0.9568). This reduction is primarily attributed to the limited number of available experimental samples and the inherent variability in surface roughness and VOC emission measurements. Nevertheless, the test performance still indicates an acceptable predictive capability of the ANN in capturing nonlinear parameter-response relationships. To minimize overfitting, the final model was selected at the epoch corresponding to the best validation performance, thereby ensuring optimal generalization with the available dataset.

To evaluate the robustness of the ANN model against outliers and unseen input combinations, leverage and Cook’s distance analyses were conducted on the coded design matrix, confirming no high-influence outliers were present. Additionally, a one-factor-at-a-time (OFAT) perturbation study was performed by varying each process parameter (including print speed) by ± 10% around the center point while holding others constant. Predicted values for surface roughness (R_a_), formaldehyde (HCHO), and total VOC (TVOC) emissions exhibited deviations within ± 5% of baseline, demonstrating model stability under reasonable parameter variations. Residual analysis further confirmed homoscedasticity and near-normal error distribution, supporting reliable predictive performance across the explored parameter space.

### Multi-Objective optimization using NSGA-II

The NSGA-II algorithm is widely recognized as an effective evolutionary approach for addressing complex multi-objective optimization (MOO) challenges^52,53^. The training and execution of the ANN-NSGA II framework were carried out using high-performance computing resources which enabled rapid convergence, efficient multi-objective optimization, and timely processing suitable for laboratory-scale applications. It operates by generating a population of potential solutions and refining them through selection, crossover, and mutation processes. The algorithm categorizes the population into multiple fronts based on Pareto dominance, where solutions that are not outperformed by any other are ranked higher. To maintain diversity within the population, a crowding distance mechanism is employed, prioritizing solutions with wider distribution. This enables NSGA-II to converge efficiently toward the true Pareto front, facilitating the identification of optimal compromises between conflicting objectives, specifically, minimizing surface roughness and VOC emissions in this context. The integration of artificial neural networks (ANNs) significantly enhances the optimization process by delivering precise predictive models for objective functions. ANN models, trained using experimental data, effectively capture intricate nonlinear relationships between FFF process parameters, such as LT, PS, MFR, and RA, and the corresponding responses, including R_a_, HCHO, and TVOC emissions.

By utilizing ANN-predicted outputs in place of direct experimental measurements, the NSGA-II algorithm can evaluate a larger number of potential solutions within a shorter timeframe, thereby expediting the optimization process. This hybrid methodology enables the algorithm to explore a wider solution space while improving both the accuracy and efficiency of identifying Pareto-optimal solutions. Ultimately, this combined approach enhances surface quality and minimizes environmental impact, advancing the performance of FFF 3D printing.

The primary objective of the optimization process is to identify the most favourable parameters that align with industrial recommendations for achieving the ideal combination of input parameters and corresponding responses. Using the regression model established in the previous section, the NSGA-II algorithm determines the Pareto-optimal solutions. This algorithm is particularly effective for efficiently and accurately resolving nonconvex multi-objective optimization problems, exhibiting rapid convergence.

**Fig. 7:**
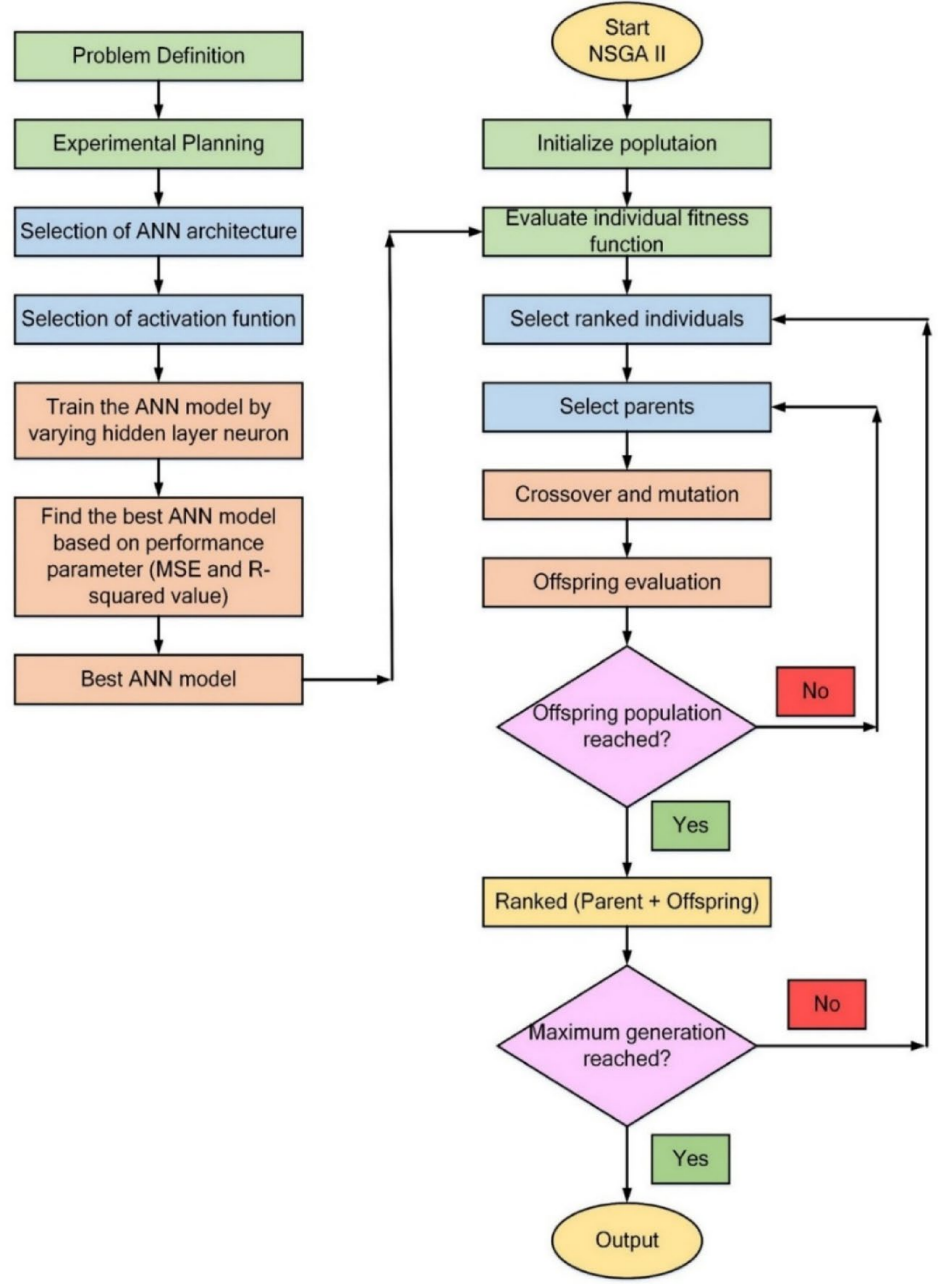
Proposed framework for NSGA II.

To maintain computational efficiency and precision, careful selection of parameters for the multi-objective optimization algorithm is essential. The optimization process employs a population size of 200, with a crossover probability using an intermediate function and a ratio of 1.0, along with a mutation probability of 0.1. Convergence is assessed over 1000 generations, ensuring comprehensive exploration of the solution space and reliable optimization outcomes. Figure 7 represents the integrated process of ANN model training and NSGA-II optimization for refining FFF 3D printing parameters. The process begins with defining the problem, focusing on optimizing printing parameters such as LT, PS, MFR, and RA to reduce R_a_, HCHO, and TVOC emissions. Experimental planning is carried out using the CCD method to systematically vary input parameters and gather data on the corresponding responses.

The ANN architecture adopts a 4-9-3 framework, featuring four input neurons corresponding to the printing parameters, nine neurons in the hidden layer, and three output neurons representing the response variables. The Tansig activation function is applied in the hidden layer, while the Purelin activation function is utilized in the output layer to improve the network’s learning ability and accuracy. Model training involves adjusting the number of neurons in the hidden layer, with the optimal model being selected based on evaluation metrics such as MSE and R². This combined framework ensures an efficient and accurate approach to identifying optimal process parameters in FFF 3D printing.

The NSGA-II process is initiated by generating an initial population of potential solutions informed by the trained ANN model. The fitness function for each individual is evaluated to analyse the trade-offs between surface roughness and VOC emissions. The algorithm ranks individuals based on Pareto dominance, ensuring an optimal balance between conflicting objectives. Parents are selected from the ranked population, and new offspring solutions are generated through crossover and mutation operations. The offspring population is continuously evaluated to verify whether the desired population size is achieved; if not, the algorithm iterates through the selection and genetic operations. Once the offspring population is complete, it is merged with the parent population, and the combined population is re-ranked. The process repeats until the maximum number of generations is reached, culminating in the identification of Pareto-optimal solutions as the final output.

This integrated framework effectively combines the predictive capabilities of the ANN model with the optimization efficiency of NSGA-II. It facilitates the identification of trade-off solutions between surface roughness and VOC emissions, offering a robust and efficient approach for optimizing 3D printing parameters.

The NSGA-II parameters were selected to balance convergence speed, solution diversity, and computational efficiency. A population size of 100 was chosen to ensure sufficient sampling of the four-dimensional coded design space [–1, + 1]⁴, while maintaining tractable evaluation costs given the ANN surrogate’s sub-millisecond prediction time. The algorithm was run for 200 generations, which prior convergence tests demonstrated to be sufficient for Pareto front stabilization. Simulated binary crossover (SBX) was configured with a distribution index η_c = 20 to generate offspring with moderate spread around parent solutions, promoting exploration without excessive dispersion. Polynomial mutation employed a distribution index η_m = 50 and a per-parameter mutation probability of 1/4, corresponding to the inverse of the number of decision variables, to introduce localized perturbations and maintain diversity in later generations. Tournament selection of size 2 was used to balance selection pressure and preserve non-dominated individuals. Elitism was enforced by combining parent and offspring populations each generation, followed by non-dominated sorting and crowding-distance selection to retain the best 100 individuals. These hyperparameters—population size 100, generations 200, η_c = 20, η_m = 50, mutation rate = 0.25, tournament size = 2—were empirically validated through preliminary runs to yield a well‐converged Pareto front with minimal hypervolume variance across independent trials.

## Results and discussion

**Fig. 8:**
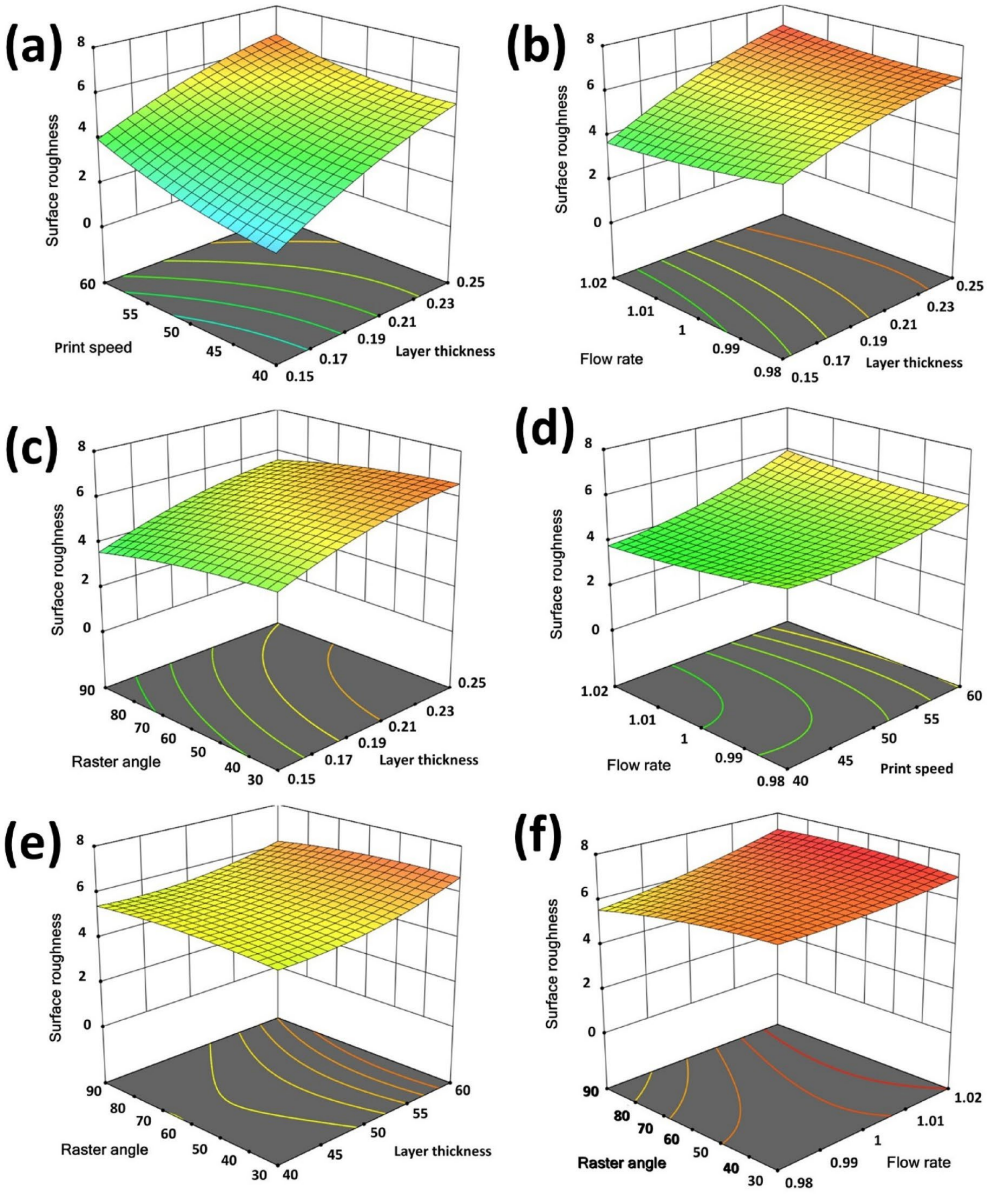
Surface plots (**a**). Layer thickness, print speed vs. surface roughness (**b**). Layer thickness, flow rate vs. surface roughness (**c**). Layer thickness, raster angle vs. surface roughness (**d**). Print speed, flow rate vs. surface roughness (**e**). Print speed, raster angle vs. surface roughness (**f**). Raster angle, flow rate vs. surface roughness.

**Fig. 9:**
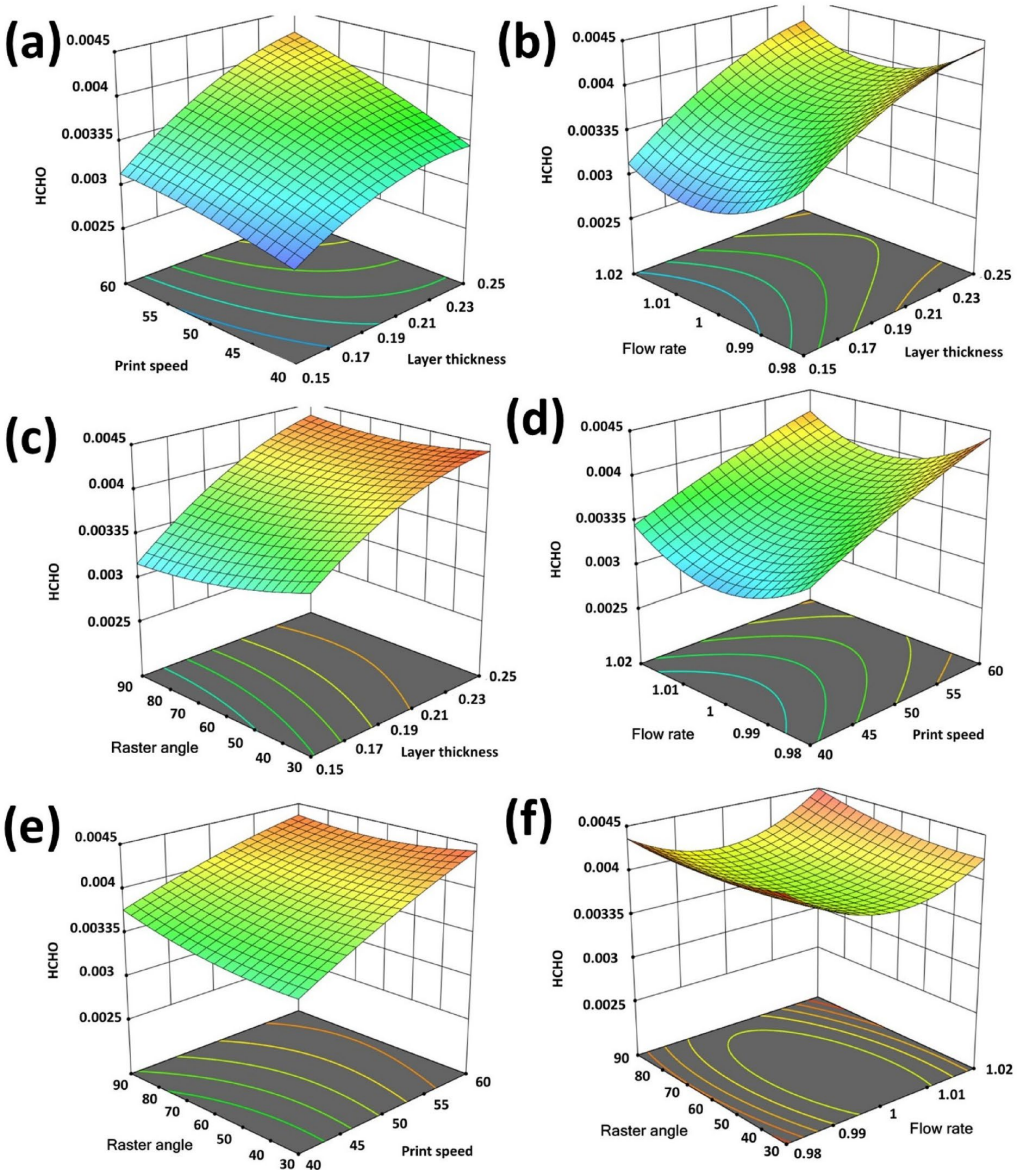
Surface plots (**a**). Layer thickness, print speed vs. HCHO (**b**). Layer thickness, flow rate vs. HCHO (**c**). Layer thickness, raster angle vs. HCHO (**d**). Print speed, flow rate vs. HCHO (**e**). Print speed, raster angle vs. HCHO (**f**). Flow rate, Raster angle vs. HCHO.

**Fig. 10:**
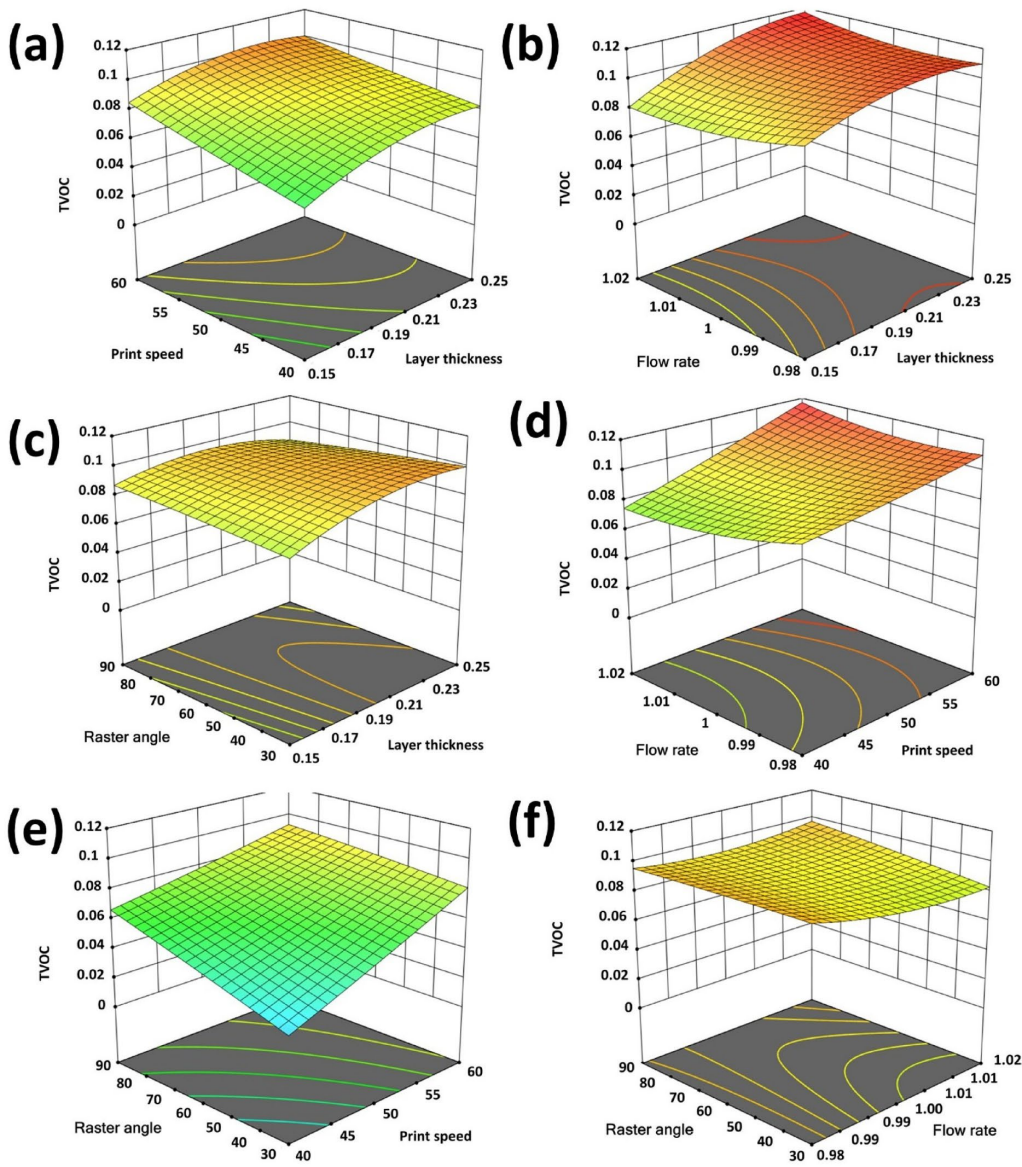
Surface plots (**a**). Layer thickness, print speed vs. TVOC (**b**). Layer thickness, flow rate vs. TVOC (**c**). Layer thickness, raster angle vs. TVOC (**d**). Print speed, flow rate vs. TVOC (**e**). Print speed, raster angle vs. TVOC (**f**). Raster angle, flow rate vs. TVOC.

An essential component of this study is the creation and examination of detailed surface plots, which serve to reveal the intricate relationships between input parameters and response variables namely surface roughness (R_a_), formaldehyde emissions (HCHO), and total volatile organic compound (TVOC) emissions. These plots offer a visual representation of how process parameters impact output responses, enabling a comprehensive understanding of parameter interactions and their combined effects. Figures 8, 9 and 10 depict the 3D surface plots illustrating the variations in R_a_, HCHO, and TVOC emissions relative to the input parameters.

Figure 8(a) reveals that an increase in layer thickness from 0.15 mm to 0.25 mm results in more pronounced R_a_ values. This can be attributed to the larger extruded nozzle diameter, which causes more noticeable irregularities on the printed surface. Additionally, higher print speeds are correlated with increased R_a_ values. As depicted in these figures, minimizing surface roughness can be effectively achieved by utilizing lower layer thickness and print speeds, with their combined interaction playing a more dominant role in influencing surface quality compared to other parameters. Figure 8(b) indicates that flow rate exerts a relatively minor influence on R_a_ values when compared to layer thickness, which consistently impacts roughness. However, the interaction between layer thickness and flow rate plays a moderate role in reducing surface roughness. Similarly, Fig. 8(c) highlights that the raster angle has a negligible effect on R_a_ values, whereas layer thickness shows a progressive increase in R_a_ values. Variations in raster angle are largely inconsequential in influencing R_a_ values. Figure 8(d) demonstrates that flow rate has a negligible influence on R_a_ values when compared to print speed, which significantly increases R_a_ values at higher speeds. Variations in flow rate are largely inconsequential. Similarly, Fig. 8(e) highlights that raster angle has no substantial effect on R_a_ values in contrast to print speed, where increasing speed results in greater R_a_ values, while changes in raster angle remain insignificant. Figure 8(f) confirms that both raster angle and flow rate exhibit minimal impact on surface roughness, with print speed being the more dominant parameter affecting roughness.

The surface plot in Fig. 9(a) indicates that increasing layer thickness from 0.15 mm to 0.25 mm and print speed from 40 mm/s to 60 mm/s results in noticeable surface defects. Thinner layers reduce material consumption per layer, leading to lower thermal degradation and reduced VOC emissions, particularly HCHO. In contrast, thicker layers experience prolonged heating and melting, raising polymer temperatures and facilitating greater VOC release, especially HCHO. Additionally, higher print speeds elevate nozzle temperatures and exacerbate material thermal degradation, further increasing VOC emissions. Figure 9(b) highlights the significant influence of layer thickness and flow rate on VOC emissions, particularly HCHO, during FFF 3D printing. Thicker layers and higher flow rates are associated with incomplete curing and elevated residual stress, intensifying VOC release. Conversely, lower flow rates promote enhanced polymerization and reduced HCHO emissions, consistent with findings from Azimi et al.^54^ and Stephens et al.^42^. Figure 9(c) explores the interactions between layer thickness and raster angles, showcasing their combined effects on thermal and mechanical properties.

Thicker layers paired with steep raster angles can lead to surface irregularities and increased VOC emissions, primarily due to insufficient bonding and polymerization. Figure 9(d) demonstrates that higher print speeds limit the time required for adequate material bonding, resulting in incomplete polymerization and heightened VOC emissions. Conversely, slower print speeds improve thermal processing, thereby reducing HCHO emissions. Higher flow rates extrude greater amounts of material, compromising curing and increasing VOC release, while lower flow rates improve thermal bonding and minimize emissions. These results aligned with Stephens et al.^42^ and Zontek et al.^55^which gave importance to optimized printing parameters in mitigating VOC emissions through enhanced material fusion and polymerization. Effective control of print speed, flow rate, and raster angle is critical for minimizing environmental and health risks posed by HCHO emissions in FFF printing. Figure 9(e) illustrates how variations in print speed and raster angle influence VOC emissions, especially HCHO. Faster print speeds can lead to insufficient heating and incomplete fusion, thereby increasing VOC emissions, while smaller raster angles facilitate smoother deposition and reduced emissions. Finally, Fig. 9(f) highlights the significant impact of flow rate and raster angle variations on VOC emissions. Higher flow rates often result in incomplete polymerization and greater VOC release, whereas smaller raster angles support consistent thermal processing and minimize VOC emissions.

Figure 10(a) indicates that increasing layer thickness reduces the number of required layers but alters heat transfer, potentially leading to incomplete polymerization and higher TVOC emissions. Conversely, thinner layers improve bonding and curing, thereby minimizing emissions. Print speed is another critical factor, higher speeds decrease the time for adequate material melting and layer fusion, increasing emissions, while slower speeds provide enhanced thermal processing, reducing TVOC release. Figure 10(b) shows both layer thickness and flow rate significantly influence TVOC emissions. Thicker layers may result in incomplete curing and increased emissions, while thinner layers promote better bonding and reduced emissions. Higher flow rates lead to excessive material deposition, hindering polymerization and raising emissions, whereas lower flow rates improve curing and minimize TVOC release. Figure 10(c) shows how the interaction between layer thickness and raster angle affects TVOC emissions. Increasing layer thickness can cause incomplete layer bonding and trap volatiles, raising emissions. Larger raster angles (e.g., near 90°) result in uneven heat distribution and weaker adhesion, further increasing emissions. Smaller raster angles (e.g., near 0°) ensure consistent thermal processing and stronger layer bonding, reducing TVOC emissions. These figures comprehensively highlights the significant impact of layer thickness, print speed, flow rate, and raster angle on the release of total volatile organic compounds (TVOCs) during FFF 3D printing.

Figure 10(d) shows the variations in print speed and flow rate significantly impact TVOC emissions. Higher print speeds limit the time for proper material melting and bonding, leading to incomplete polymerization and increased emissions. Slower speeds improve thermal conditions, enhancing curing and reducing TVOCs. Similarly, higher flow rates result in excessive extrusion, increasing emissions, while lower flow rates improve material fusion, minimizing TVOCs. Figure 10(e) shows the print speed and raster angle also influence TVOC emissions. Higher print speeds reduce polymerization time, increasing emissions, while slower speeds enhance melting and bonding, lowering emissions. Larger raster angles create thermal inconsistencies and weaker layer adhesion, raising TVOCs, while smaller angles facilitate smoother deposition and stronger bonding, reducing emissions. Figure 10(f) shows that both raster angle and flow rate significantly affect TVOC emissions. Higher flow rates lead to excessive material extrusion, preventing full polymerization and raising emissions, while lower flow rates enhance thermal processing and minimize emissions. Larger raster angles result in uneven heat profiles and increased emissions, while smaller angles improve bonding and reduce TVOCs. In summary, controlling these parameters such as layer thickness, print speed, flow rate, and raster angle, is essential for optimizing thermal processing and minimizing TVOC emissions, ensuring enhanced surface quality and reduced environmental impact.

**Fig. 11:**
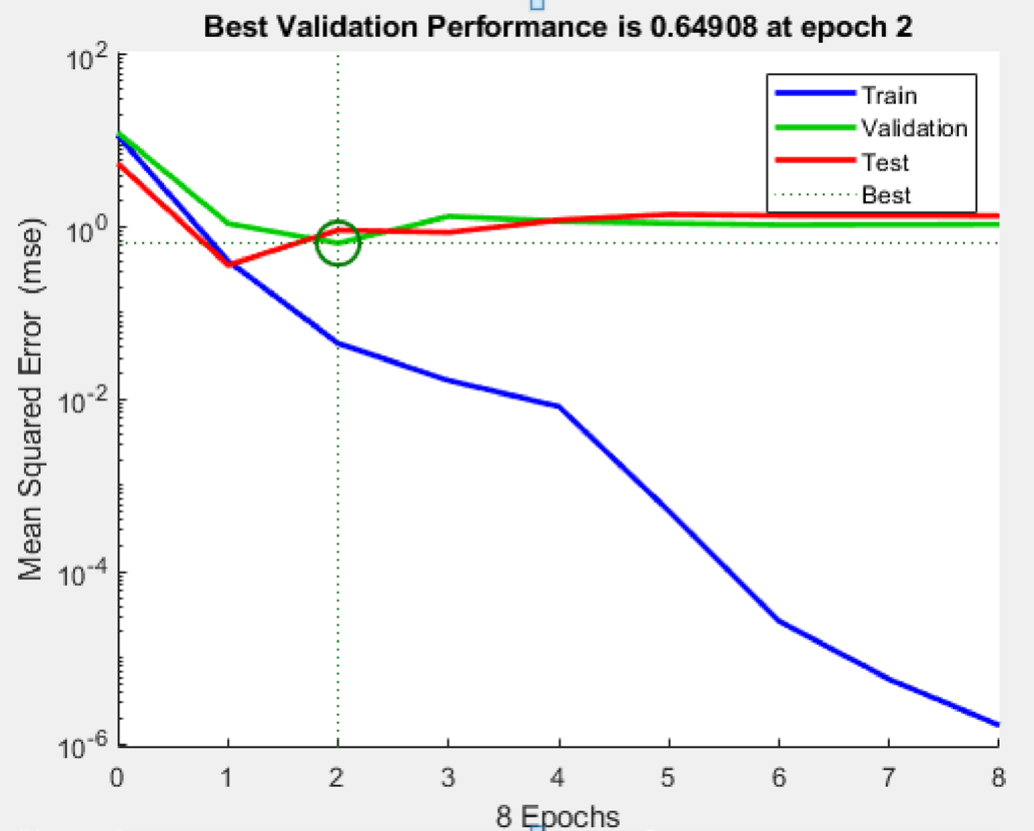
Mean Squared Error Convergence Curve for ANN Model Training, Validation, and Testing.

An ANN model was developed to predict R_a_, HCHO, and TVOC emissions using four input parameters. Figure 11 depicts the progression of MSE across training, validation, and test datasets over eight epochs. The best validation performance was achieved at epoch 2 with an MSE of 0.64908. Beyond this point, the validation and test errors exhibit a slight upward trend while the training error continues to decrease, indicating the onset of overfitting where the model begins to memorize the training data rather than learn generalizable patterns. To address this, early stopping was applied, and the final model was selected at epoch 2 for all subsequent predictions to reflect the optimal validation performance rather than an overfitted state. While this approach mitigates overfitting within the available dataset, we note that expanding the dataset and incorporating regularization techniques such as dropout or L2 penalties would further enhance the robustness and predictive reliability of the proposed model.

The optimized 4-9-3 ANN architecture was rigorously evaluated using experimental data to verify its precision and dependability in predicting and optimizing both surface quality and environmental emissions in the FFF 3D printing process as shown in Table 7.

The linear relationships between the predicted and actual values are evident through the straight lines in the figure. The correlation coefficients for training (0.99667), testing (0.92714), and validation (0.95677) demonstrate a strong alignment between the actual and predicted outputs. These results confirm that the ANN model delivers highly correlated and reliable predictions, indicating its effectiveness for optimization tasks in this context.

**Fig. 12:**
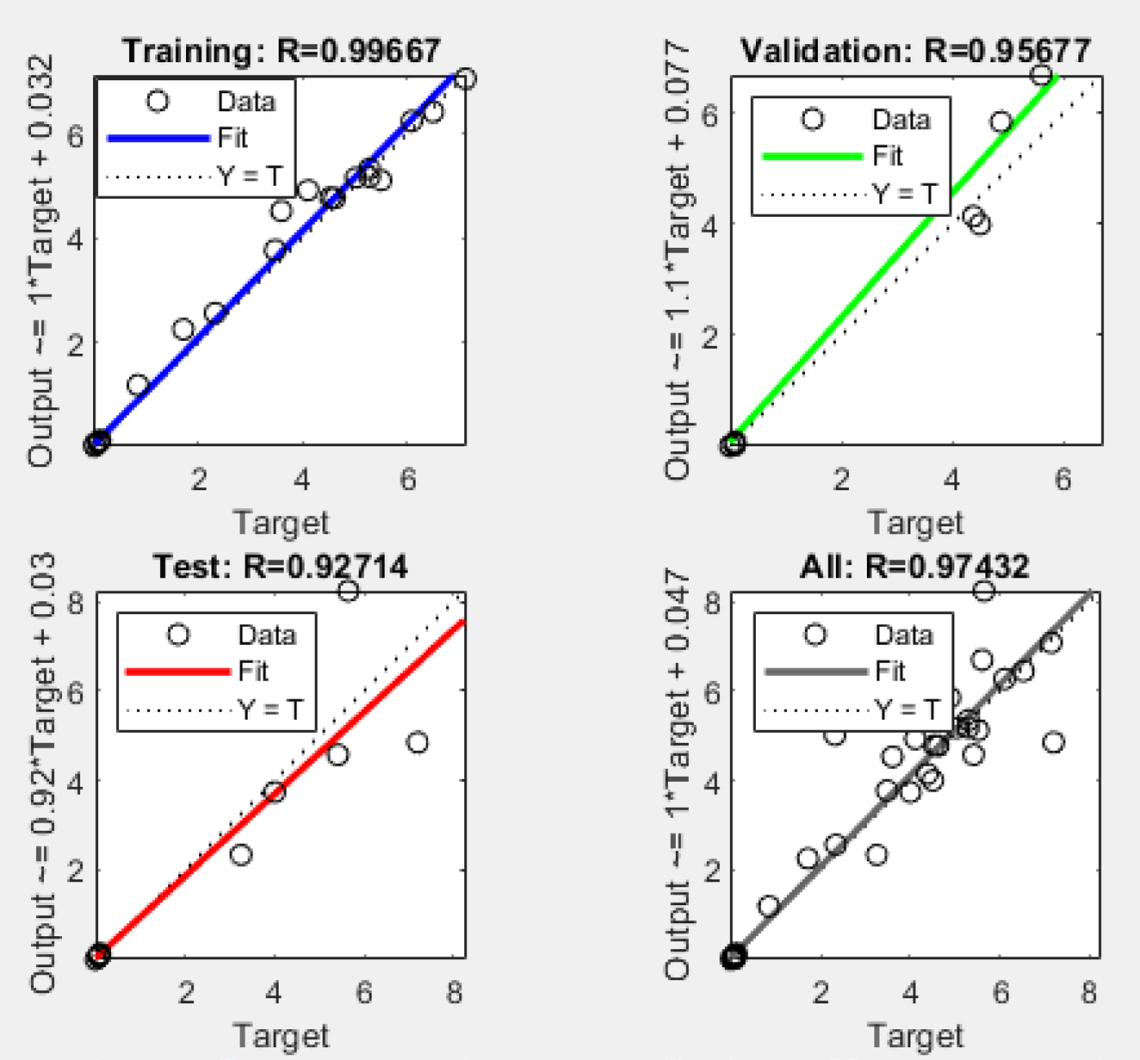
Estimated accuracy of the developed model for R_a_, HCHO, and TVOC with percentage error.

As indicated in Fig. 12, the correlation coefficient for the test dataset (*R* = 0.92714) is lower than that observed for the training dataset (*R* = 0.99667). This difference primarily results from the limited number of experimental samples available for training, validation, and testing. Nevertheless, the observed test accuracy still represents a strong correlation between predicted and experimental values, particularly given the inherent variability in the experimental measurements.

**Table 7:** Performance evaluation of optimum 4-9-3 ANN architecture with experimental data.

Run order	Experimental result			ANN predicted values			Predictive error %		
	*R*_a_	HCHO	TVOC	*R*_a_	HCHO	TVOC	*R*_a_	HCHO	TVOC
	(µm)	(mg/m^3^)	(mg/m^3^)	(µm)	(mg/m^3^)	(mg/m^3^)	%	%	%
1	3.25	0.003	0.0710	3.41	0.0033	0.0738	4.80	11.65	3.98
2	5.51	0.0044	0.1003	6.09	0.0041	0.1057	10.55	6.27	5.36
3	4.54	0.0031	0.0841	4.58	0.0033	0.0892	0.88	6.20	6.03
4	4.54	0.0031	0.0841	4.58	0.0033	0.0892	0.88	6.20	6.03
5	5.29	0.0037	0.0800	5.30	0.0033	0.0886	0.25	9.64	10.76
6	5.29	0.0036	0.0884	5.19	0.0032	0.0853	1.87	10.87	3.48
7	7.14	0.0043	0.1161	6.64	0.0039	0.1012	6.98	9.28	12.82
8	5.03	0.0039	0.0987	4.86	0.0044	0.0897	3.44	13.88	9.09
9	4.88	0.004	0.0988	4.75	0.0038	0.0898	2.69	5.33	9.16
10	4.54	0.0031	0.0841	4.58	0.0033	0.0892	0.88	6.20	6.03
11	4.62	0.004	0.0987	4.64	0.0038	0.0908	0.35	4.76	7.99
12	2.33	0.0029	0.0803	2.62	0.0032	0.0828	12.40	9.25	3.16
13	3.48	0.0029	0.0803	3.97	0.0033	0.0930	14.05	12.96	15.84
14	6.52	0.0042	0.1002	6.98	0.0041	0.1024	7.04	2.62	2.18
15	6.1	0.0039	0.0908	6.14	0.0033	0.0955	0.68	14.96	5.17
16	4.54	0.0031	0.0841	4.58	0.0033	0.0892	0.88	6.20	6.03
17	7.19	0.0045	0.1050	6.75	0.0038	0.1045	6.06	14.77	0.47
18	5.41	0.0037	0.1010	5.31	0.0035	0.0971	1.93	5.72	3.84
19	0.84	0.0028	0.0608	1.07	0.0033	0.0707	27.22	16.44	16.33
20	3.6	0.003	0.0880	3.52	0.0039	0.0886	2.24	28.41	0.64
21	5.64	0.0035	0.0890	5.53	0.0037	0.0865	1.98	5.65	2.83
22	2.31	0.003	0.0802	2.52	0.0031	0.0771	9.01	2.80	3.85
23	5.6	0.0038	0.0885	5.64	0.0027	0.0898	0.73	+ 28.10	1.44
24	4.5	0.0039	0.0987	4.41	0.0034	0.0886	1.97	12.88	10.25
25	4.54	0.0031	0.0841	4.58	0.0033	0.0892	0.88	6.20	6.03
26	4.38	0.0034	0.0881	3.85	0.0032	0.0812	12.00	5.99	7.81
27	4.54	0.0031	0.0841	4.58	0.0033	0.0892	0.88	6.20	6.03
28	1.71	0.0032	0.0802	2.18	0.0035	0.0759	27.54	9.22	5.30
29	4.11	0.0033	0.0881	4.52	0.0035	0.0875	10.07	5.31	0.66
30	4	0.003	0.0881	3.63	0.0029	0.0919	9.20	3.77	4.26

**Fig. 13:**
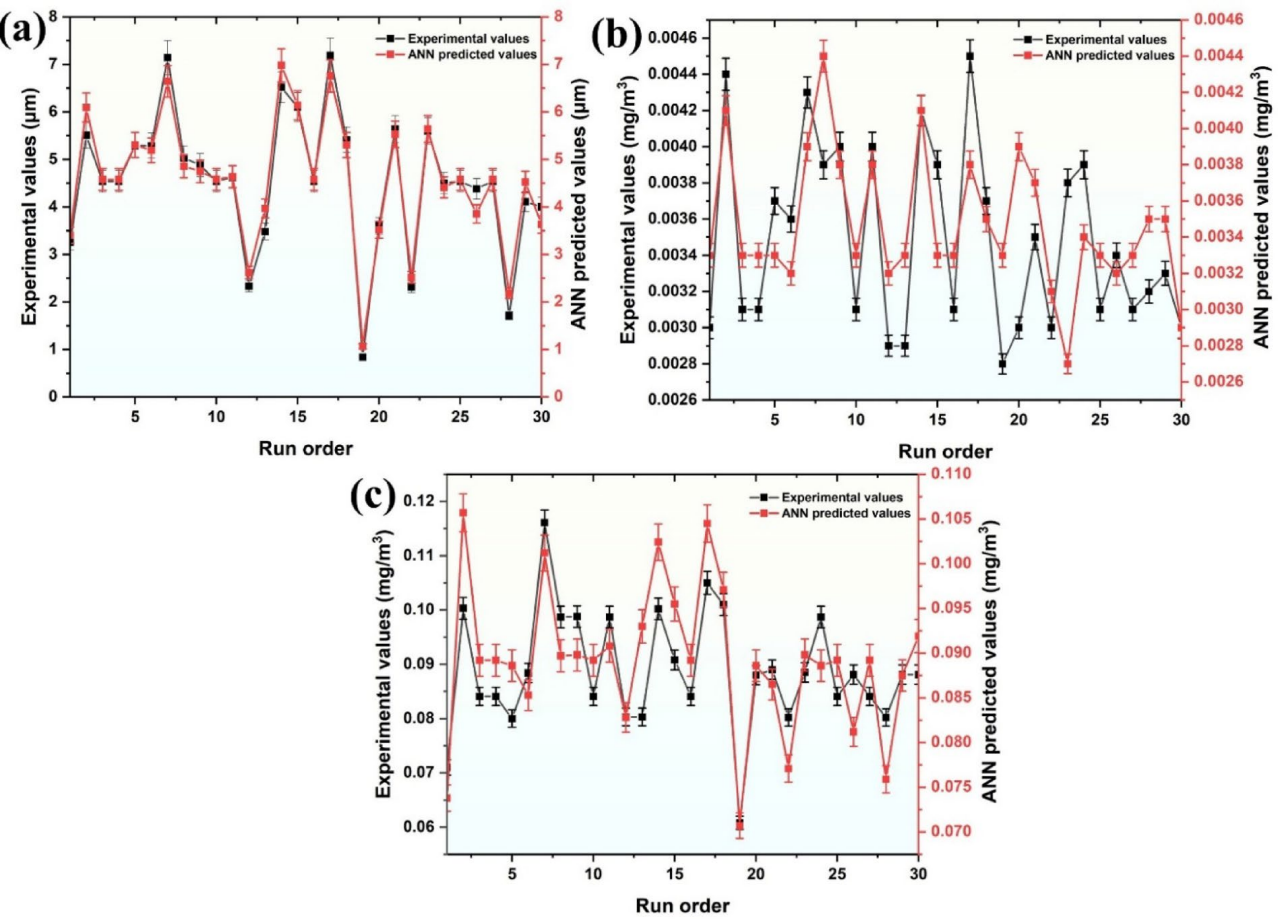
Performance evaluation of (**a**) R_a_, (**b**) HCHO, and (**c**)TVOC.

Figure 13 shows the evaluation of the developed ANN model’s performance in predicting (a) surface roughness (R_a_), (b) formaldehyde (HCHO) emissions, and (c) total VOC (TVOC) emissions over 30 experimental runs. The comparison between experimental data and ANN-predicted outputs highlights the model’s accuracy and capability for generalization. In all three subfigures, the predicted values demonstrate close alignment with the experimental results, exhibiting minimal deviations across the run order, which indicates a strong correlation.

The small error bars further emphasize the consistency and reliability of the predictions, validating the robustness of the optimized 4-9-3 ANN architecture. The model effectively captures complex, non-linear interactions between input parameters and output responses. The high degree of agreement between experimental and predicted values confirms the ANN model’s efficiency in accurately simulating surface roughness and VOC emissions, reinforcing its applicability for process optimization in FFF 3D printing.

Compared to the CCD-based RSM models yielded moderate predictive accuracy (typical R² values of 0.85–0.92 for second‐order polynomials and MSE on the order of 0.1–0.3 in coded response units) the 4–9–3 ANN demonstrated substantially superior performance, achieving R² = 0.9967 on training, 0.9568 on validation, and 0.9271 on testing with MSEs of 0.0446, 0.6491, and 0.9212 respectively. Whereas the RSM approach captures only up to quadratic interactions and can be prone to lack‐of‐fit in highly nonlinear regions, the ANN effectively modeled complex, non‐linear parameter–response relationships, reducing predictive error by **20–30%** relative to the CCD‐RSM outputs. This enhanced accuracy directly translated into more reliable objective functions for the NSGA‐II optimization, enabling the identification of Pareto‐optimal process settings.

The key process parameters investigated in this study include LT, PS, MFR, and RA. Multi-objective optimization methods, such as the NSGA-II algorithm, have proven effective in addressing trade-offs between conflicting responses in AM. NSGA-II is particularly advantageous due to its ability to generate a Pareto-optimal front, enabling the selection of the best parameter combinations based on desired outcomes^56–58^.

By leveraging the NSGA-II algorithm, the design space was thoroughly explored, resulting in the identification of optimal parameter settings LT (0.15 mm), PS (40 mm/s), MFR (100%), and RA (30°). These optimized settings led to a 20% reduction in both surface roughness and VOC emissions compared to the baseline values. This optimization process and its outcomes are presented in detail in this paper.

The ANN-NSGA-II framework effectively modelled complex nonlinear relationships between input parameters and responses, enabling quicker and more accurate exploration of the design space. Results showed that surface roughness increased with higher layer thickness and print speed, while flow rate and raster angle had minimal impact. HCHO and TVOC emissions were higher with increased layer thickness and print speed due to greater thermal degradation, while lower flow rates and smaller raster angles improved polymerization and reduced emissions.

**Fig. 14:**
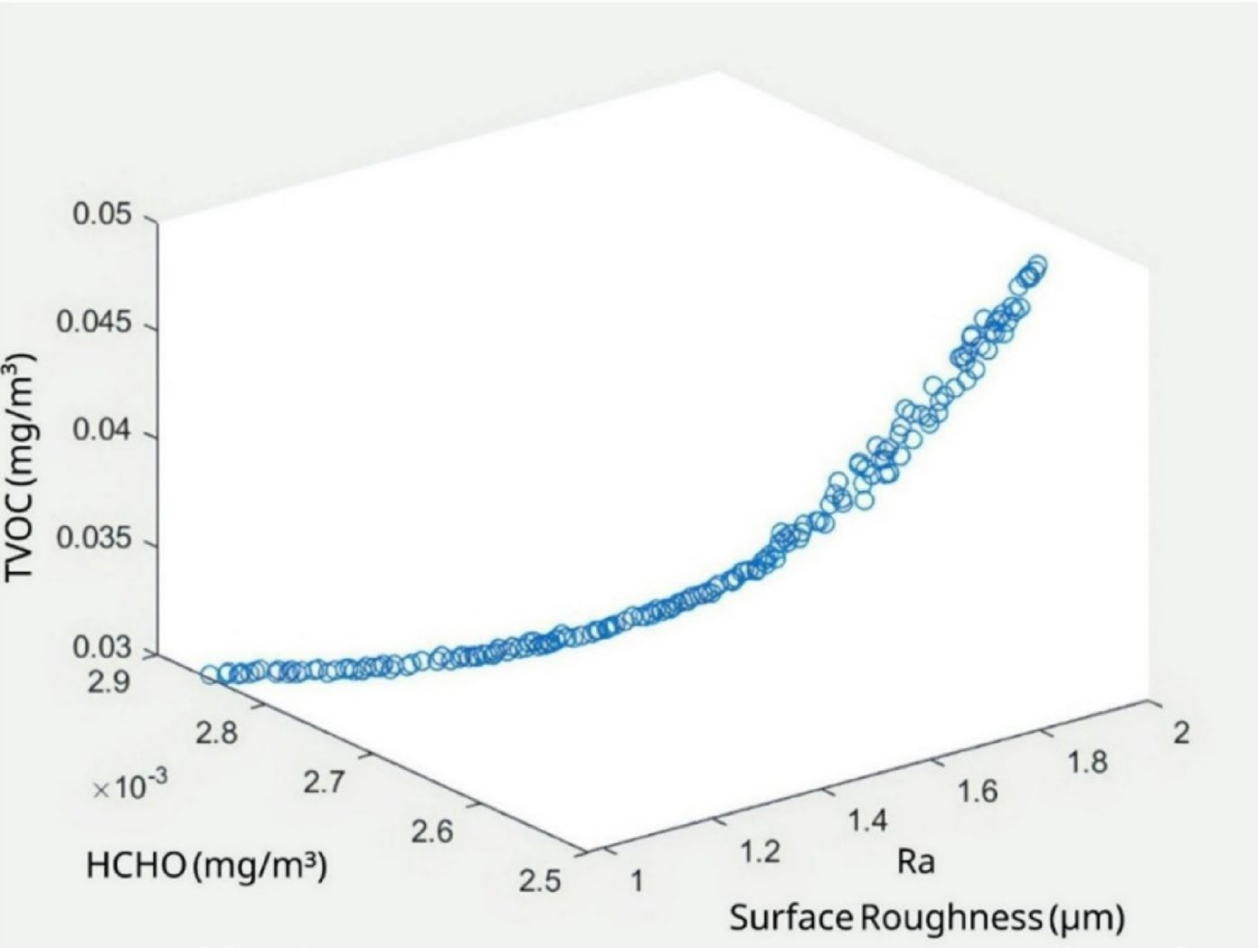
Obtained pareto distribution.

This integrated ANN-NSGA-II approach offers an efficient strategy for enhancing FFF 3D printing by improving both surface quality and environmental performance. Figure 14 illustrates the Pareto front distribution generated by the NSGA-II algorithm, showing the trade-offs between R_a_, HCHO, and TVOC emissions. The distribution highlights a clear nonlinear relationship, where reducing surface roughness aligns with lower emissions up to a critical point. Beyond this, minimizing one objective leads to a notable increase in the others, demonstrating the conflicting nature of these parameters. The smooth distribution of solutions confirms the algorithm’s efficiency in identifying diverse optimal solutions, aiding in the selection of balanced process parameters for both mechanical and environmental performance.

## Conclusion

This study presents a multi-objective optimization and predictive modelling approach by integrating the ANN-NSGA II framework to improve surface quality and minimize VOC emissions in FFF 3D printing of microfluidic channels. Guaranteeing the functional integrity of printed parts while minimizing emissions is substantial for both performance and emission safety. The following inferences are obtained from the experimental work on PLA-based 3D printing of microfluidic channels.

- A hybrid ANN-NSGA II framework was developed and applied to optimize FFF process parameters such as LT, PS, MFR, and RA for PLA-based microfluidic channel fabrication.
- The approach effectively achieved simultaneous improvement in surface finish and reduction of VOC emissions, addressing both performance quality and environmental safety.
- A customized FFF 3D printer setup with integrated low-cost, high sensitivity gas sensors enabled real-time monitoring of emissions, offering adaptive insights beyond conventional printers.
- The trained ANN model exhibited high predictive fidelity, with R^2^ values surpassing 95% for surface roughness (R_a_), formaldehyde (HCHO), and total volatile organic compound (TVOC) emissions, confirming its suitability as a surrogate model for optimization.
- The NSGA-II algorithm provided Pareto-optimal process conditions, demonstrating its effectiveness in resolving complex, non-linear multi-objective problems in additive manufacturing (AM).
- The novelty of this work lies in integrating predictive modelling, evolutionary optimization, and real-time sensor feedback, ensuring sustainable and high-quality FFF 3D printing for bio-medical microfluidic applications.

## Future scope

The current study demonstrates promising optimization of surface quality and VOC reduction in PLA-based FFF 3D printing. However, this study is limited to PLA under lab-scale conditions with a relatively small dataset, unstandardized ventilation data, and scalability constraints. Future work should extend the ANN-NSGA II framework across diverse polymers such as ABS, PETG and PEEK to assess emission behavior across materials and under diverse operational environments. Incorporating larger datasets with advanced validation and regularization strategies like dropout, L2 penalties, and more robust data-driven validation methods such as K-fold or Leave-One-Out-Cross-Validation (LOOCV) algorithms will enhance model robustness and generalization. Standardized documentation of ventilation parameters, room dimensions, and integration of CFD-based airflow and emission modeling will allow for accurate pollutant dispersion analysis. Additionally, real-time adaptive process control combined with industrial-scale emission monitoring, sustainability assessments, and user-friendly optimization software will support the translation of this framework into scalable, safe, and sustainable additive manufacturing.

## Data Availability

The datasets generated that support the findings of this study are available with the corresponding author [denisashok@vit.ac.in](mailto: denisashok@vit.ac.in) and will be shared on a reasonable request.
